# A model of estrogen-related gene expression reveals non-linear effects in transcriptional response to tamoxifen

**DOI:** 10.1186/1752-0509-6-138

**Published:** 2012-11-08

**Authors:** Galina Lebedeva, Azusa Yamaguchi, Simon P Langdon, Kenneth Macleod, David J Harrison

**Affiliations:** 1 Centre for Synthetic and Systems Biology, University of Edinburgh, CH Waddington Building, the Kings Buildings, Mayfield Road, EH9 3JD, Edinburgh, UK; 2 School of Informatics, University of Edinburgh, 10 Crichton Street, Edinburgh, UK; 3 Division of Pathology, IGMM, University of Edinburgh, Western General Hospital, Edinburgh, EH4 2XU, UK; 4 Medical and Biological Sciences Building, University of St Andrews, North Haugh, St Andrews, Fife, KY16 9TF, UK

**Keywords:** Estrogen receptor, Cancer, Tamoxifen, SERM, Mathematical model, Global sensitivity analysis

## Abstract

**Background:**

Estrogen receptors alpha (ER) are implicated in many types of female cancers, and are the common target for anti-cancer therapy using selective estrogen receptor modulators (SERMs, such as tamoxifen). However, cell-type specific and patient-to-patient variability in response to SERMs (from suppression to stimulation of cancer growth), as well as frequent emergence of drug resistance, represents a serious problem. The molecular processes behind mixed effects of SERMs remain poorly understood, and this strongly motivates application of systems approaches. In this work, we aimed to establish a mathematical model of ER-dependent gene expression to explore potential mechanisms underlying the variable actions of SERMs.

**Results:**

We developed an equilibrium model of ER binding with 17β-estradiol, tamoxifen and DNA, and linked it to a simple ODE model of ER-induced gene expression. The model was parameterised on the broad range of literature available experimental data, and provided a plausible mechanistic explanation for the dual agonism/antagonism action of tamoxifen in the reference cell line used for model calibration. To extend our conclusions to other cell types we ran global sensitivity analysis and explored model behaviour in the wide range of biologically plausible parameter values, including those found in cancer cells. Our findings suggest that transcriptional response to tamoxifen is controlled in a complex non-linear way by several key parameters, including ER expression level, hormone concentration, amount of ER-responsive genes and the capacity of ER-tamoxifen complexes to stimulate transcription (e.g. by recruiting co-regulators of transcription). The model revealed non-monotonic dependence of ER-induced transcriptional response on the expression level of ER, that was confirmed experimentally in four variants of the MCF-7 breast cancer cell line.

**Conclusions:**

We established a minimal mechanistic model of ER-dependent gene expression, that predicts complex non-linear effects in transcriptional response to tamoxifen in the broad range of biologically plausible parameter values. Our findings suggest that the outcome of a SERM’s action is defined by several key components of cellular micro-environment, that may contribute to cell-type-specific effects of SERMs and justify the need for the development of combinatorial biomarkers for more accurate prediction of the efficacy of SERMs in specific cell types.

## Background

Estrogen receptor mediated signalling is highly implicated in the growth regulation of hormone dependent cancers, such as breast, ovarian and uterine cancer [1,2].

According to the current concept, estrogen receptor alpha (ER) belongs to the class of nuclear hormone receptors that act as transcription factors, controlling expression of genes that regulate cell survival and proliferation. The molecular mechanisms of ER-induced transcriptional activation include binding of ER with its natural ligand 17β-estradiol, upon which the receptor undergoes a conformational change, which favors its dimerisation and binding to the specific promoter sequences within DNA - so called Estrogen Response Elements (ERE). Binding of an ER-ligand complex to ERE initiates a further cascade of processes, resulting in the assembly on the promoter of a multi-component ER-transcription complex. This involves combinatorial binding of multiple co-regulator proteins, which can either activate or suppress transcription of ER-responsive gene [3,4]. The composition of the transcription complex is believed to be cell and tissue-specific [5-7].

Current therapeutic strategies in the treatment of ER-positive cancers include administration of drugs that act as estradiol competitors, displacing hormone in its binding site on ER (e.g., tamoxifen, raloxifene). These drugs were initially designed as anti-estrogens with a view to suppress pro-mitogenic actions of estradiol in cancer cells [8,9]. However, as revealed by further studies, the developed compounds were capable of exhibiting both antagonist and agonist activities, depending on the type of tissue, in which they acted, that led to redefinition of these drugs as Selective Estrogen Receptor Modulators (SERMs) [10-12]. In particular, tamoxifen can act as an ER antagonist in breast cancer cells [13], but, at the same time it has a partial agonistic effect in the endometrium, resulting in endometrial hyperplasia and an increased risk of cancer [5,10].

Multiple studies have been undertaken to tackle the molecular mechanisms, underlying the dual agonist/antagonist action of SERMs and the associated emergence of drug resistance. Significant efforts have focused on deciphering the role of transcriptional co-regulators [14,15]. Other studies have attempted to define specific gene expression signatures, associated with sensitivity or resistance to SERMs [16-18]. Recent study of Ross-Innes et al. [19] highlighted the importance of genome-wide ER-binding events, and presented convincing evidence that specific FOX-1 mediated "reprogramming" of ER binding with DNA in breast tumors significantly contributes to the variation in clinical outcome.

Despite significant progress in understanding the system of ER-related signalling, and advances in deciphering the molecular components involved in cellular response to estrogens and SERMs, the mechanisms responsible for cell type specific and patient-to-patient variability in responding to SERMs still remain not fully understood.

The high level of combinatorial complexity of early transcriptional events, initiated by ER binding with ERE, as well as an intricate web of signalling pathways associated with ER (e.g., growth factor and cytokine-related signalling), makes it impractical to analyse the ER signalling network based solely on intuitive reasoning. This strongly motivates the application of mathematical abstraction and computational approaches. However, before now, surprisingly little has been done to address the complexity of ER-related signalling with modelling approaches, and mathematical models of estrogen related processes are still sparse. In 1993 Dove and Schonenberger [20] presented a simple equilibrium model of ER binding with estrogen and DNA that was successfully applied to explain different types of experimentally registered estrogen dose–response curves. Recently Tyson et al. highlighted the importance of developing a comprehensive mathematical model of the estrogen signalling network in breast epithelial cells, and outlined the roadmap for the development of such a model [1].

In this study we aimed to develop a simple mechanistic model for estrogen-induced gene expression, focusing on the very first steps of cellular response to estrogens and SERMs, mediated by ER binding with DNA. We established a detailed equilibrium model of ER binding with two types of ligands (natural hormone and a SERM) and DNA ERE, and linked it to a simplified ODE model of transcription and translation of an estrogen-responsive gene. We parameterised the model on the broad range of literature available experimental data, including *in vitro* data on ER binding with ligands and DNA, and the reporter gene expression data, obtained in HEK 293/hERα cell line, treated with 17β-estradiol and tamoxifen.

We demonstrate that the analysis of the steady state solution of the developed model provides a plausible mechanistic explanation for the dual agonist/antagonist action of tamoxifen in the HEK 293/ERα cell line used for model calibration. We further explore the applicability of our conclusions to a more general context (i.e. to other cell types) by running global sensitivity analysis and exploring the behaviour of the model solution in the wide range of biologically plausible parameter values. We demonstrate that the magnitude of ER-dependent transcriptional response to tamoxifen is controlled in a complex non-linear way by several key parameters, including expression level of ER, background concentration of 17β-estradiol, amount of estrogen-responsive elements and the capacity of ER-tamoxifen complexes to induce transcription (e.g. via recruiting specific co-regulators of transcription). We discuss the potential applicability of these findings in the context of biomarker research.

## Results and discussion

### Mathematical model

Our model of estrogen-induced gene expression consists of three main blocks, corresponding to three key types of processes included in the system: (1) interaction of ER with ligands; (2) binding of receptor-ligand complexes with DNA ERE and (3) ER-induced transcription and translation.

**Figure 1:**
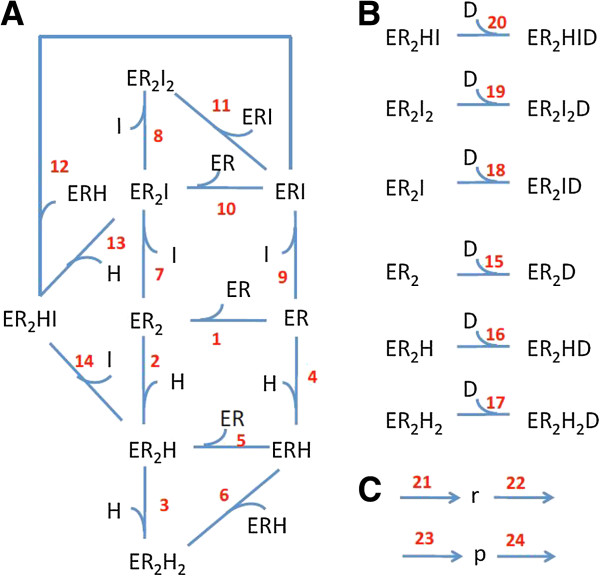
**General scheme of the processes included in the model of ER-dependent gene expression.** (**A**) Reactions of ER dimerisation and binding with two types of ligands - natural hormone (H) and an inhibitor (I); (**B**) Binding of dimeric forms of receptor-ligand complexes with ERE; (**C**) Synthesis and degradation of mRNA (r) and protein (p). The detailed description of the scheme is given in the text.

The general scheme of the model is presented in Figure 1. Figure 1A describes the processes of ER dimerisation and binding with ligands, and was constructed based on the following reasoning. As suggested by structural studies [21] both ligand binding and dimerisation activities of the ER monomer are located within its ligand-binding domain (LBD), with ligand binding cavity and ER dimerisation surface spatially separated from each other. Therefore, in terms of our model we considered them as two distinct binding sites within ER - one for ligand binding, and one for binding with other monomers. Under these assumptions we considered the possibility of formation of nine different receptor-ligand complexes, resulting from interaction between ER monomers and two different receptor ligands - a natural agonist hormone 17β-estradiol (H), and a SERM, which acts as a competitive inhibitor (I), occupying ER hormone binding site. The lower part of the scheme shown in Figure 1A depicts various receptor-ligand complexes and their transformations, resulting from ER dimerisation and interaction with the hormone (reactions 1–6), while the upper part describes competitive binding of an anti-estrogenic drug (I) to ER (reactions 7–9) and dimerisation of the ER-inhibitor complexes (reactions 10 and 11). In our model we consider a possibility of heterodimer formation (ER_2_HI), with one ER monomer bound to the hormone, and another one - to an inhibitor (reactions 12–14).

We assume that binding of a hormone or an inhibitor by an ER monomer can affect ER dimerisation, that may result in differences in kinetic constants assigned to stages 1, 5, 6, 10, 11 and 12. We also assume that monomeric (ER) and dimeric (ER_2_) forms of the receptor differ by their affinity to ligands.

It must be noted, that experimental confirmation of the existence of each of the receptor-ligand binding combinations included in the model poses significant challenges, because of practical difficulties associated with discriminating between the experimentally registered effects of similar binding complexes (e.g., ER_2_H and ER_2_H_2_). However, the existence of free (unliganded) ER dimer (ER_2_), and its capacity to stimulate ER-dependent transcription have been confirmed in a number of experimental studies [22-25]. For example, Tamrazi et al. [23] confirmed the possibility of ER_2_ formation and demonstrated that addition of ligands affected dimer stability in dose-responsive manner, with maximal effect observed when receptor is fully saturated with ligand. This provides an indirect evidence for existence of ER_2_H and ER_2_I complexes. Indeed, if both ER_2_H_2_ and ER_2_ do exist, than ER_2_H has to be included into consideration based on the step-wise mechanism of binding/dissociation of two hormone molecules to/from ER_2_H_2_. Similar reasoning can be used to justify existence of ER_2_I complexes. The existence of ER_2_HI complex can be questionable, but we included it in the model since its occurrence is theoretically possible.

Figure 1B describes binding of different forms of ER_2_ dimers to ERE sequences of DNA (D) (reactions 15–20). Here we assume that various dimeric forms of ER-ligand complexes have different affinities for ERE (i.e. the values of K_d_ for reactions 15–20 differ from each other). In our model we do not consider binding of the ER monomers with ERE, since this process was shown to be much less efficient than binding of ER dimers [26-28]. In particular, Carlsson et al. (1995) [27] reported 20-fold lower association rate constant of the hormone-receptor complex to ERE at low concentrations of ER, when the predominant form of ER is a monomer, as compared to higher concentrations (ER > 12 nM), when the majority of ER present as homodimers. Metzger et al. (1995) also reported that the wild type ER binds *in vitro* to ERE as a dimer, irrespective of the presence or absence of estrogen [28].

Finally, reactions 21 and 23 shown in Figure 1C depict the processes of transcription (synthesis of mRNA, r) and translation (synthesis of the protein, p), whereas reactions 22 and 24 account for degradation of RNA and protein, respectively. In our model we assume that the rate of mRNA synthesis is proportional to the level of active ER-ERE transcription complexes.

The full ODE system describing the dynamics of the system shown in Figure 1 would include 16 ODEs, four algebraic equations and 45 independent parameters (see Additional File 1). The reliable identification of all kinetic parameters of such ODE system would represent a challenging task due to the lack of available dynamic data. Importantly, the majority of existing experimental studies into estrogen-induced transcription and effects of SERMs focus on exploring various kinds of dose–response relationships, quantifying the response of the experimental system to specific ER ligands, in the broad range of ligand concentration. The typical experimental setup includes several hours pre-incubation of either purified estrogen receptors, or ER expressing cells with relevant receptor ligands (hormones, SERMs), followed by the measurement of specific receptor-ligand binding or by quantification of cellular response to the treatment (e.g. expression of specific proteins). Such a significant time of pre-incubation with the ligands (from a few hours *in vitro* up to 72 h *in vivo* and cell-based systems) allows assuming that the experimental system reaches either an equilibrium (*in vitro*) or a steady state (*in vivo*).

Thus, to be able to utilize the available data on equilibrium binding and dose–response curves for model parameterisation, we needed to consider the model at an equilibrium or a steady state.

It is worth noting, that equilibrium approximation of ER binding with ligands and DNA ERE may in fact be sufficient for a simplified description of ER dependent gene expression, since the system allows separation of the time scales between faster ligand binding processes and slower gene expression events. Thus we established an equilibrium model of ER binding with two competing ligands and DNA. A similar equilibrium approach was previously applied by Dove et al. [20] to describe ER binding with a single ER agonist and DNA, that allowed successful reproduction and explanation of different patterns of dose response curves measured *in vivo.* Our developed equilibrium model was then linked to a simplified kinetic model of transcription and translation of an ER-dependent gene, and the steady state solution of the whole model has been analysed. Further details of model development are given in Methods.

### Model parameterisation

The values of model parameters have been estimated based on experimental data available from the literature. It is worth noting, that although there exist many available estimates for some of the kinetic constants of ER interaction with ligands, there is a high level of diversity in the available data - e.g., the parameter values are often reported for various forms of the receptor (ERα, ERβ), extracted from different organisms (mice, calf, rat, human), and measured under a variety of experimental conditions (e.g. at different temperature). Importantly, most estimates do not allow discrimination between the ligand binding parameters for monomeric and dimeric forms of ER. Moreover, these estimates often have been obtained with the use of different methods. This results in a significant discrepancy in the reported values (e.g. for receptor binding with 17β-estradiol the reported K_d_ estimates range from 0.1 [29] to 0.5 [30] and 1 nM [31], depending on the method and the receptor type used. To reduce the level of parametric uncertainty in our model, we only used those data sets, which were measured for human ERα (hER). Importantly, where possible, we have not just re-used the reported values, but re-fitted the reported experimental curves with our model, to achieve the best match between theoretical and experimental trajectories. This in most cases resulted in some level of re-adjustment of the initial parameter estimates, taken from the literature.

**Figure 2:**
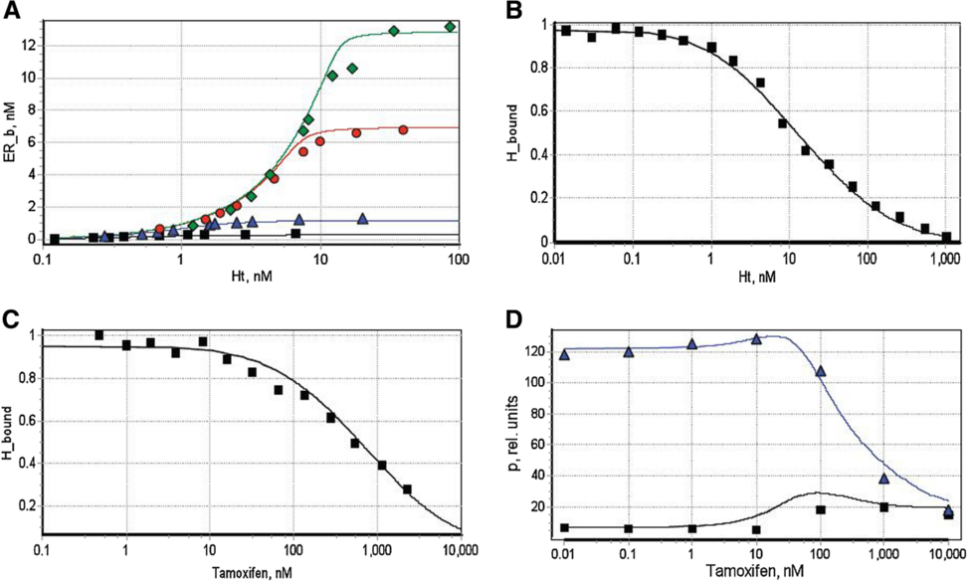
**Fitting the model of ER-dependent gene expression to multi-level experimental data.** (**A**) The equilibrium model of ER binding with the hormone was fitted against the data on equilibrium binding of recombinant hERα with labelled estradiol (17β -[6,7 -^3^H(N)] estradiol), registered at four different concentrations of hERα: 0.3 (black square); 1.2 (blue triangle); 7 (red circle); and 13 (green diamond) nM. The data were taken from [33]. ER_b is the concentration of ER binding sites, bound with hormone; Ht -total concentration of labelled estradiol. (**B**) and (**C**): The equilibrium model was used to fit the data on competitive binding of ligands with ER in the presence of labelled 17β-estradiol. The competitive ligands were: unlabelled 17β-estradiol (**B**) and tamoxifen (**C**). The experimental data were taken from [34]. (**D**) The steady state protein expression (p) was fitted to the data on reporter gene expression (ALP) in HEK293/hERα cell line treated with tamoxifen (black line) and tamoxifen+0.5 nM 17β-estradiol (blue line); data taken from [38]. Points correspond to experimental data, lines – to theoretical curves. More details on the fitting procedure and model calibration can be found in the Additional File 2.

Model parameterisation included three main stages, in accordance with the model architecture, and following the strategy of step-by-step integration of multi-level experimental data within kinetic models [32]. The model has been decomposed into smaller sub-systems, which have been parameterised separately with the use of suitable experimental data (Figure 2). For example, to identify parameters of ER interaction with ligands we fitted the model against published *in vitro* data on binding of purified hER with hormone [33] and tamoxifen [34]. To evaluate K_d_s for ER interaction with DNA we used the data on hER binding to ERE DNA [35-37]. The estimated parameters have then been integrated into the whole system. The parameters of the full system have been further fine-tuned, via fitting the full model against the data set, obtained with the use of an *in vivo* cellular assay [38].

Further details on calibration of separate blocks of the model can be found in the Additional File 2. The identified parameter values are given in Tables 1 and 2.

**Table 1:** Parameters of ER binding with ligands and DNA ERE

**Reaction**	**K**_**d**_	**Value in the model, nM**	**Existing experimental estimates, nM**	**Reference**
ER+ER=ER_2_	K1	25	>50	[73]
ER_2_+H=ER_2_H	K2	0.2	0.16	[23]
ER_2_H+H=ER_2_H_2_	K3	0.1	-	
ER+H=ERH	K4	0.25	0.9	[31]
ERH+ER=ER_2_H	K5^*^	20	50	[73]
ERH+ERH=ER_2_H_2_	K6^*^	8	4	[33]
			20	[73]
ER_2_+I=ER_2_I	K7	10	-	
ER_2_I+I=ER_2_I_2_	K8	150	220	[31]
ER+I=ERI	K9	30	-	
ERI+ER=ER_2_I	K10^*^	8.3	< K5	[23]
ERI+ERI=ER_2_I_2_	K11^*^	41.5	-	
ERH+ERI=ER_2_HI	K12^*^	26.67	-	
ER_2_I+H=ER_2_HI	K13	0.8	-	
ER_2_H+I=ER_2_HI	K14^*^	40	-	
ER_2_+D=ER_2_D	K15	8	40	[35]
			2.5	[36]
ER_2_H+D=ER_2_HD	K16	2.4	-	
ER_2_H_2_+D=ER_2_H_2_D	K17	1.2	1.8 ±0.6	[36]
			2.0±0.3	[27];
			10	[35]
ER_2_I+D=ER_2_ID	K18	50	-	
ER_2_I_2_+D=ER_2_I_2_D	K19	87	14	[37]
ER_2_HI+D=ER_2_HID	K20	10-20	-	

Parameters marked by (*) are dependent on the values of others and have been calculated as described in Methods.

**Table 2:** Parameters of transcription and translation

**Process**	**Rate constant**	**Value in the model, s**^**-1**^	**Existing experimental estimates, s**^**-1**^	**Reference**
mRNA synthesis induced by ER_2_-hormone complexes	*k*_*sr*_	0.195	0.125-0.2	[74]
mRNA synthesis induced by ER_2_-inhibitor complexes	*k*_*sr,i*_	0.02	-	
mRNA synthesis induced by ER_2_-hormone-inhibitor complexes	*k*_*sr,hi*_	0.05	-	
mRNA synthesis induced by ER_2_	*k*_*sr,b*_	0.001	-	
mRNA degradation	*k*_dr_^*^	(0.0025)^*^	0.001-0.003	[74]
			1.5×10^-4^-0.005	[75]
Protein synthesis	*k*_sp_^*^	(0.001)^*^	3×10^-4^ - 0.03	[76]
Protein degradation	*k*_dp_^*^	(2×10^-5^)^*^	1.4×10^-5^ -3×10^-5^	[76]
Constant ratio $\frac{k_{\mathrm{dp}}k_{\mathrm{dr}}}{k_{\mathrm{sp}}}$	*κ*	5×10^-5^		

Parameters marked (*) could not be evaluated independently. Use of the dose dependence data only allowed estimation of the ratio *κ*, as defined in equation (22). The given values are realistic estimates of *k*_*dr*_*, k*_*sp*_ and *k*_*dp*_ chosen to satisfy the equation for *κ* (see Additional File 3 for further details).

Despite the broad diversity of experimental data used for calibration of its separate blocks, the resulting model was capable of qualitatively matching the tamoxifen dose-dependence patterns of ER-induced gene expression in a reporter cell line, and allowed realistic description of a number of important regulatory phenomena, known for ER-dependent transcription. Moreover, further analysis of the model provided some valuable insights into potential mechanisms underlying dual agonism/antagonism action of tamoxifen in various cell types, as outlined in the next sections.

### Description of the cooperativity of the ER ligand binding

The positive cooperative nature of ER binding with 17β-estradiol has been reported in multiple studies [29,33,35,39], that revealed a transition from non-cooperative to positive cooperative binding, associated with gradual increase in ER concentration from low to high levels.

The level of cooperativity is commonly characterised with the use of Hill coefficient *n*_*H*_, estimated from fitting experimental binding curves with the empirical Hill equation:

$$B=\frac{{\left[L\right]}^{n_{H}}}{{\left[K_{A}\right]}^{n_{H}}+{\left[L\right]}^{n_{H}}}$$

where *B* is a fraction of the receptor binding sites occupied by ligands, *L* - concentration of free ligand, *K*_*A*_ - ligand concentration, causing occupation of half of the binding sites, and *n*_*H*_ - Hill coefficient.

**Figure 3:**
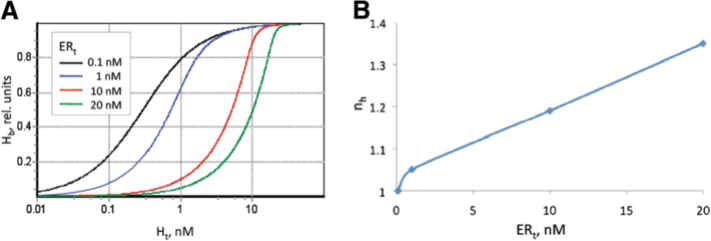
**Cooperativity of ligand binding as observed in the equilibrium model of ER binding with hormone.** (**A**) Theoretical dependence of hormone binding on the total concentration of the hormone, calculated for 1; 5; 10 and 20 nM of ER. H_b_ – fraction of the receptor binding sites occupied by hormone. (**B**) Dependence of the apparent Hill coefficient (n_H_) on ER concentration. To estimate the values of n_H_, the theoretical curves shown in Figure 3A were presented in traditional Hill coordinates (bound *vs* unbound ligand) and then fitted with the Hill equation.

Importantly, our model does not make use of the Hill equation and *n*_*H*_ coefficient to account for the cooperativity of ER binding with ligands. Instead we mechanistically describe the interaction of ER monomers with other ER monomers and ligands, that allows flexible adjustment of the model behaviour and its cooperativity level in accordance with ER concentration. It is worth noting, that reported *n*_*H*_ values for hER binding with 17β-estradiol normally do not exceed 1.4-1.5 [33,35,39]. As can be seen from Figure 3, our model reproduces a realistic range of experimentally observed cooperativity, with estimated *n*_*H*_ values ranging from 1.0 to 1.4.

### Transcriptional response to tamoxifen in HEK293/hERα cells has a complex structure

The data on tamoxifen effects in HEK293/hERα cells [38], used for initial calibration of our model (Figure 2D), clearly revealed a dual agonist/antagonist activity of the drug. In particular, in the absence of 17β-estradiol (lower curve), tamoxifen acted as a partial agonist, stimulating the ER-dependent expression of the reporter gene (ALP) in the range of concentrations > 10 nM, that resulted in a 4-fold increase in ALP expression as compared with the basal level, and up to 10% of the maximal transcriptional response caused by 17β-estradiol. Interestingly, in the presence of 0.5 nM 17β-estradiol, in the tamoxifen concentration range > 100 nM, the drug acted as as a pure anti-estrogen, causing a suppression of the reporter gene expression. However in the range of concentrations 1–100 nM, tamoxifen was not only ineffective in suppressing protein expression, but even had some stimulatory effect, manifested as a slight increase in ALP production at 10–50 nM of tamoxifen.

Note, that similar stimulatory effects of tamoxifen on ER dependent transcriptional activation and cell proliferation have been reported in other studies [11,40]. For example, McDonnel et al. [11] reported partial agonism of tamoxifen in HepG2 cells, transfected with the estrogen-responsive C3-Luc reporter gene along with an ERα expression vector. At concentrations 1–100 nM tamoxifen stimulated expression of the reporter gene, up to 30% of the level induced by estradiol. Higher concentrations of tamoxifen led to suppression of the gene expression. Similar dose dependences were observed with the use of cell-proliferation assays, demonstrating that in certain cell lines and in the concentration range 1–100 nM, tamoxifen was not just ineffective in suppressing cell proliferation, but in fact stimulated these processes [41-43].

Since our model was capable of qualitatively matching the observed effects, we further sought to analyse the obtained solution to get an insight into potential molecular mechanisms underlying these phenomena.

**Figure 4:**
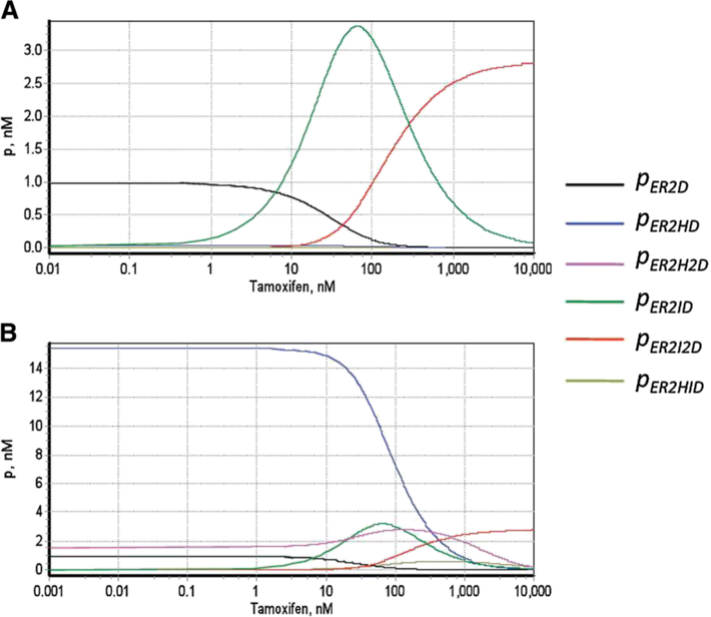
**Analysis of the composition of transcriptional response to tamoxifen in HEK 293/hERα cell line.** The curves represent partial transcriptional responses caused by various forms of transcription complexes assembled at ERE, calculated as tamoxifen dose dependences of the following terms: (1) *p̅ *_*ER*2*D*_ = *k*_*sr*,*b*_*ER*_2_*D*/*κ*; (2) *p̅ *_*ER*2*HD*_ = *k*_*sr*,*h*_*ER*_2_*HD*/*κ*; (3) *p̅ *_*ER*2*H*2*D*_ = *k*_*sr*,*h*_*ER*_2_*H*_2_*D*/*κ*; (4) *p̅ *_*ER*2*ID*_ = *k*_*sr*,*i*_*ER*_2_*ID*/*κ*; (5) *p̅ *_*ER*2*I*2*D*_ = *k*_*sr*,*i*_*ER*_2_*I*_2_*D*/*κ*;  (6) *p̅ *_*ER*2*HID*_ = *k*_*sr*,*hi*_*ER*_2_*HID*/*κ*. Concentration of 17β-estradiol was set equal to 0 (**A**) and 0.5 nM (**B**) in accordance with the reference experiment [38].

In our model the steady state level of ER-dependent protein expression (*p̄*) is fully defined by the partial transcriptional activation effects caused by various forms of transcription complexes assembled at ERE, as specified in equations 22–24 (see Methods). To estimate the contribution of each of these complexes into resulting protein expression we generated separate trajectories of tamoxifen dose dependence for each of the terms in the equations (22–23), in a wide range of tamoxifen concentrations (Figure 4). According to our analysis, in the absence of estradiol (Figure 4A), and at low tamoxifen (< 10 nM) the gene expression is maintained at some background level, provided by free receptor dimers bound with ERE (ER_2_D). In the range of 10–100 nM tamoxifen, the ER-dependent transcription is mainly driven by the ER_2_ID complexes, whose contribution gradually increases reaching a peak at about 50 nM tamoxifen and then steadily drops. At higher tamoxifen concentrations (>200 nM) the key role in transcription activation shifts to ER_2_I_2_D complexes, whose amount progressively grows with the increase of tamoxifen concentration. Thus, our analysis suggests that the partial agonism of tamoxifen in the absence of the hormone results from transcriptional activation caused by transcription complexes formed by ER_2_ bound with tamoxifen (ER_2_ID, ER_2_I_2_D).

In the presence of 0.5 nM estradiol (Figure 4B), the composition of transcriptional response to tamoxifen is more complex, since it includes stimulatory effects caused by the hormone-receptor complexes. The model predicts that at concentration lower than 50 nM, tamoxifen is unable to effectively antagonise estradiol-induced transcription, because of the high level of transcriptional stimulation, caused by ER_2_HD complexes (Figure 4B, blue curve). In the range of 10–100 nM of tamoxifen the input of ER_2_HD complexes into overall transcription gradually decreases, but at the same time there is an additional "boost" in transcriptional activation, caused by the complexes ER_2_ID and ER_2_H_2_D (Figure 4B, green and magenta curves). This may explain an agonistic effect of tamoxifen in the dose range of 10–100 nM, manifested as a characteristic "bump" on the dose response curve for protein expression (Figure 2D). At higher tamoxifen concentrations (>100 nM) the stimulatory action of hormone-receptor complexes on the transcription further drops, whereas the contribution of slower transcribing ER_2_I_2_D complexes progressively increases, until at tamoxifen > 1μM it becomes a prevailing factor. This is accompanied by a general decrease in ER-dependent protein expression to about 15% of its original level.

Thus, according to our analysis, in the HEK 293/hERα cell line, the overall transcriptional response to tamoxifen has a complex structure, defined by partial stimulatory effects, caused by various transcription complexes assembled at ERE. The composition of the response and contribution of each of the possible transcriptional complexes into overall transcription flexibly changes depending on the concentration of tamoxifen and background level of estradiol. Importantly, our analysis suggests that the observed antagonistic action of high dose tamoxifen arises from re-distribution of the balance between transcription complexes with lower and higher potency to stimulate transcription.

### Global sensitivity analysis of transcriptional response to tamoxifen

Our findings around the potential mechanism underpinning dual agonism/antagonism action of tamoxifen, presented above, have been derived from the analysis of a single model solution, obtained from fitting the model against the data on tamoxifen effects in a particular cell line (HEK293/hERα).

In order to extend these conclusions to more general cases we next sought to explore the model behaviour in a wider range of plausible parameter values. Indeed, many of the model parameters (such as background hormone concentration, ER expression level, number of ER-dependent genes, etc.) are likely to be cell-type specific and subject to biological variation. Moreover, a noticeable biological variability can be observed even within the same cell line. For example, Osborne et al. [44] reported considerable biological differences among MCF-7 breast cancer cells taken from different laboratories, including variable amounts of estrogen receptor. Such differences may cause significant variation in individual responses to anti-estrogens and therefore require additional investigation. In addition to that, little quantitative information is available about some of the processes, included in the model. For example, little is known about the interaction of ER with non- perfectly palindromic ERE, despite the predominance of non-classical EREs in endogenous genes. It is also worth noting, that many elementary reactions considered in the model cannot be studied in isolation, due to practical difficulties in experimental separation of the effects of individual reactions from the effects of others (e.g. it is hardly possible to discriminate between binding of ER_2_H and ER_2_H_2_ to ERE). These limitations hamper reliable identification of corresponding model parameters.

Thus, both cell type specific biological variability of parameter values and the parametric uncertainty associated with the lack of relevant kinetic data dictated the need to analyse the model behaviour in a wider parameter space.

To identify the key model parameters, whose variation may have the biggest impact on the transcriptional response to tamoxifen, we used global sensitivity analysis (GSA). The GSA technique allows exploration of the sensitivity of specific model outputs to simultaneous variation of multiple model parameters within broad parameter space, and has recently proven its value for the analysis of the range of biological systems [45-47].

In the current study we applied GSA technique for the analysis of the sensitivity of ER-induced transcriptional response to the variation of all model parameters, at different fixed doses of tamoxifen. As a quantitative measure of transcriptional activation we used the steady-state level of protein expression, *p̄*, as defined in the equation (22) (see Methods for further details on GSA).

**Figure 5:**
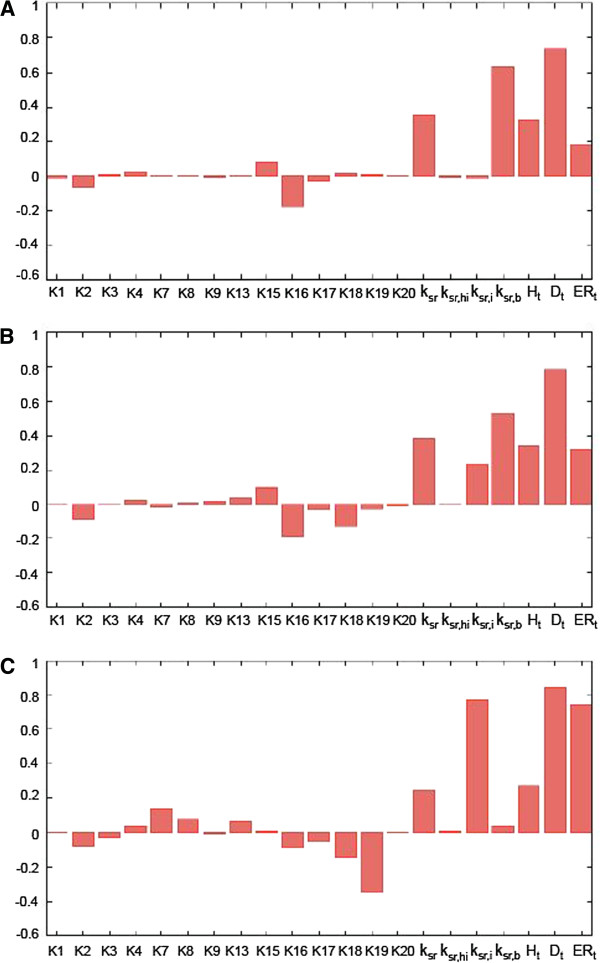
**Global sensitivity analysis of transcriptional response to tamoxifen.** Global sensitivity profile of the steady state protein expression *p̅ * was calculated for (**A**) 0.1; (**B**) 50 and (**C**) 1000 nM tamoxifen. Bars in the diagrams correspond to the PRCC sensitivity indexes calculated for each of the model parameters, as described in Methods.

Figure 5 presents the *p̄* sensitivity profiles, generated for three different concentrations of tamoxifen (see Additional File 3 for full *p̄ * sensitivity profile in the wide range of tamoxifen concentrations).

Independently of the tamoxifen concentration, the level of *p̄ * had the highest sensitivity to the total concentration of the estrogen-responsive elements ERE (D_t_), whereas the set of other top-ranked model parameters changed depending on tamoxifen concentration. In particular, in the absence and at low tamoxifen (< 0.1 nM) *p̄* was highly sensitive to the parameters *k*_*sr,b*_ and *k*_*sr*_, controlling the rate of transcription activation, induced by free ER dimers (ER_2_) and ER dimers bound to the hormone estradiol (ER_2_H, ER_2_H_2_), respectively. Relatively high sensitivity was also found for the total concentration of estradiol (H_t_) and the receptor (ER_t_), as well as for the affinity of ER_2_H complexes to DNA ERE (K_16_).

At 50 nM tamoxifen, *p̄* retained high positive sensitivity to parameters *k*_*sr,b*_ and *k*_*sr*_. At the same time it became sensitive to the rate of transcription activation (*k*_*sr,i*_) induced by the receptor-tamoxifen complexes (ER_2_I, ER_2_I_2_), and acquired negative sensitivity to the parameter K_18_, which controls binding of ER_2_I complexes to ERE. The sensitivity to the total concentration of ER (ER_t_) also significantly increased.

At high tamoxifen concentration (1000 nM), the sensitivity profile demonstrated noticeable changes as compared to lower tamoxifen concentration. In particular, *p̄* became extremely sensitive to the rate of transcriptional activation induced by tamoxifen-receptor complexes (*k*_*sr,i*_), and to the total concentration of estrogen receptor (ER_t_). It also acquired a high negative sensitivity to the parameter of ER_2_I_2_ binding to DNA (K_19_). At the same time the sensitivity to the parameters controlling transcriptional activation by free receptor dimers (*k*_*sr,b*_) and receptor-hormone complexes (*k*_*sr*_) became substantially lower. The sensitivity of *p̄* to the background hormone concentration also dropped.

Thus, the GSA revealed that out of >20 model parameters, only a few had a profound effect on the level of ER-dependent gene expression, and their ranking order was dependent on basal tamoxifen concentration. The parameters with consistently high impact in the wide range of tamoxifen concentration include: the amount of ER-dependent promoters (D_t_), estrogen receptor level (ER_t_), and background hormone concentration (H_t_). Importantly, our analysis predicts that at 1000 nM tamoxifen (that roughly corresponds to tamoxifen concentration found in tumors) the overall ER-dependent transcriptional response becomes extremely sensitive to the parameters, associated with transcriptional activation induced by tamoxifen-receptor complexes (*k*_*sr,i*_, K_19_)

### Variation of the key control parameters, identified in GSA, affects transcriptional response to tamoxifen

Next, we embarked on a more detailed investigation of how the variation of the key control parameters, identified by GSA, can affect the transcriptional response to tamoxifen. We were particularly interested in analysing these effects in a realistic concentration range of the biological components included in our model system. For this purpose, based on literature data, we estimated plausible ranges of the estrogen receptor levels, DNA ERE, hormone and drug concentration, observed in human normal and cancerous breast tissue cells (see Additional File 4 for details). In summary, the ER expression level was estimated to vary from 1pm (sensitivity cut-off for ER negative cells) to up to 300 nM in ER positive cells***,*** depending on the cell type (e.g. according to different studies wild type MCF-7 cells contain 10–50 nM ER [48,49]). Typical levels of 17β-estradiol were found to vary from as low as 30 pM in benign breast cells in postmenopausal women to as high as 1–3 nM in malignant breast tumor cells in premenopausal women [50]. Tamoxifen levels in blood plasma vary from 10–20 nM at low dose (1 mg/day) up to 200 nM at high dose tamoxifen (20 mg/day). Importantly in cancer cells tamoxifen concentrations tend to be significantly higher than in plasma and range from 100 nM to 2 μM at low and high dose tamoxifen respectively [51]. The amount of gene promoters, directly regulated by ER (D_t_), was estimated to vary in the range of 0.01-1 nM [19,52,53]

**Figure 6:**
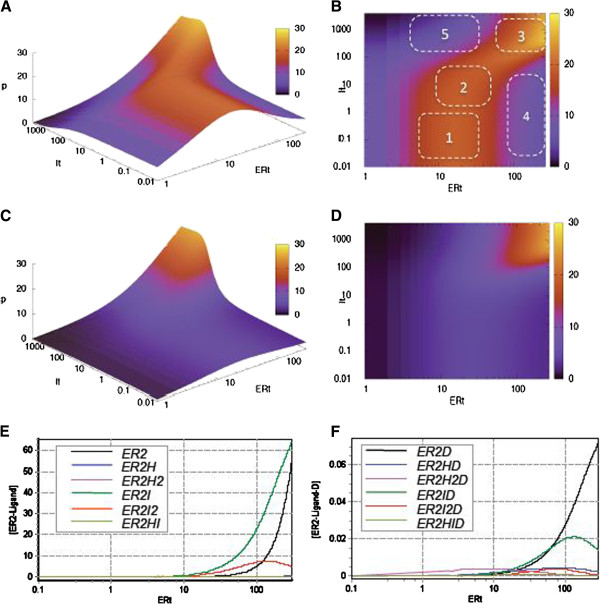
**Exploration of transcriptional response to tamoxifen in the wide range of ER concentrations.** 3D-graphs (**A**,**C**) and their 2D-projections (**B**,**D**), showing the dependence of the steady state protein expression *p̅ * on the concentration of tamoxifen and estrogen receptor. Simulations were run for 0.5 (**A**,**B**) and 0.1 nM (**C**,**D**) of 17β-estradiol. Numbers on the graph 6B correspond to the areas of high and low tamoxifen agonism, as explained in the text. (**E**) and (**F**): Concentration of different forms of receptor-ligand and transcription complexes present at ERE as a function of ER expression level. The concentration of hormone (H_t_) and tamoxifen (I_t_) were fixed at 0.5 and 100 nM respectively.

**Figure 7:**
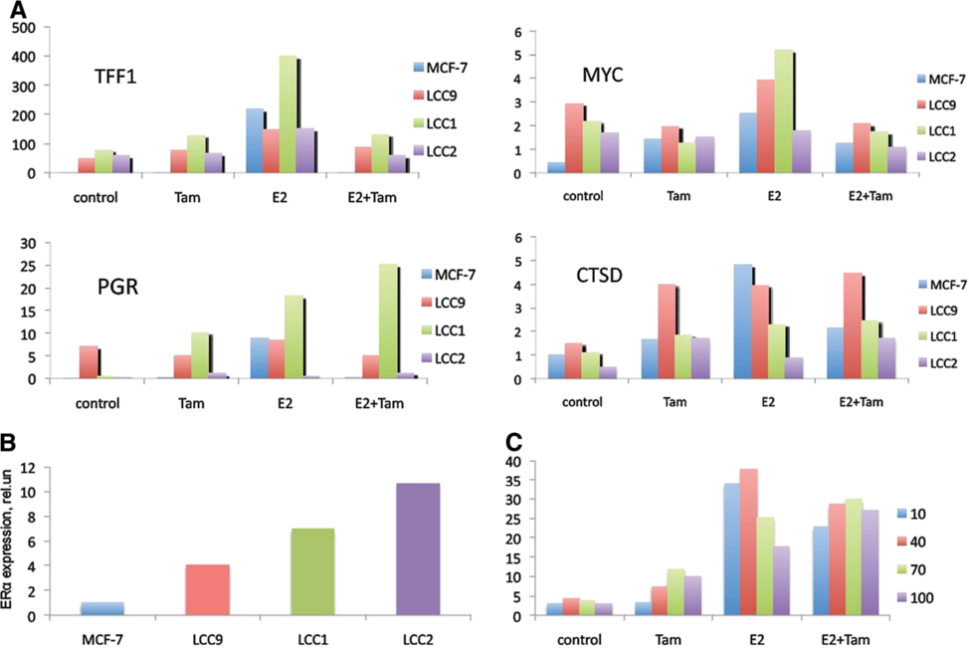
**Testing the effect of the ER expression level on the ER-dependent transcriptional activation.** (**A**) Experimental testing of the model predictions about the non-monotonous dependence of ER -induced transcriptional activation on the level of ER expression. mRNA expression levels of TFF1, PGR, MYC and CTSD in four experimental cell lines were measured by real-time RT-PCR using specific primer pairs. RNA was collected at 48 h and was extracted from either untreated (control) cells or cells treated with 1 nM 17β- estradiol (E_2_), 1 μM tamoxifen (Tam), and a combination of 1 nM E_2_ and 1 μM tamoxifen (E_2_+Tam). mRNA levels are given in arbitrary units. (**B**) Relative expression levels of ERα in wild type MCF-7, LCC1, LCC2 and LCC9 cell lines; (**C**) Theoretical expression levels of a hypothetical estrogene responsive gene, calculated as *p̅ * (see equations 22–24 in Methods) for four different total concentrations of ER: ER_t_=10; 40; 70 and 100 nM. The parameters of transcriptional regulation were set equal to those presented in Tables 1 and 2; Hormone (H_t_) and tamoxifen (I_t_) concentrations were 1 nM and 100 nM respectively. The ratio of chosen theoretical ER levels roughly corresponded to the ratio of ER expression levels in four experimental cell lines.

**Figure 8:**
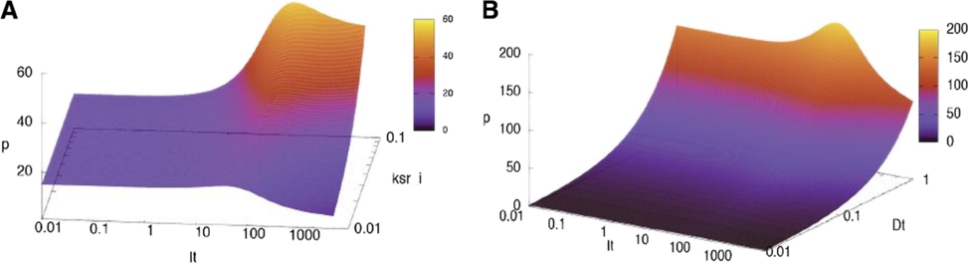
**Theoretical dependence of transcriptional response to tamoxifen on.** (**A**) the capacity of tamoxifen-receptor complexes to induce transcription (*k*_*sr,i*_); (**B**) the amount of ER-dependent genes (D_t_). The concentration of ER and 17β- estradiol were fixed at 50 and 0.5 nM respectively.

To analyse the steady state transcriptional response to tamoxifen within identified parameter ranges we generated a set of 3D graphs, demonstrating how the tamoxifen dose dependence of *p̄* changes when each of the key parameters is varied within its biologically plausible constraints (see Figures 6, 7, 8). Further we discuss our most interesting findings.

### The transcriptional response to tamoxifen depends on the level of ER expression in a non-monotonous way

#### Model predictions

Figure 6 (A-D) shows the theoretical dependence of steady state protein expression level *p̄* on the total concentration of tamoxifen and ER. Simulations were run for two different background concentrations of estradiol: higher (0.5 nM, Figure 6A,B) and lower (0.1nM, Figure 6 C,D). These roughly correspond to the upper and lower limits of 17β-estradiol in breast cancer cells in postmenopausal women [50,51].

The model suggests that the level of ER-induced protein expression is controlled by both tamoxifen and ER concentration in a complex non-linear manner. This is best illustrated by the 3D dose dependence graph, generated for higher estradiol concentration (Figure 6A), which represents a complex surface with pronounced areas of elevated protein expression. In a wide range of tamoxifen concentrations the dependence of transcriptional response on the level of ER expression is non-monotonous. In fact, even at an extremely low concentration of tamoxifen (I_t_ ≤1 nM) there is a notable maximum of transcriptional activation, observed in the range of 10–50 nM ER. The gradual elevation of tamoxifen concentration from 1 to 1000 nM results in a progressive shift of that maximum towards higher values of ER along with the increase in the amplitude of the effect, so that the maximal agonism is achieved when both tamoxifen and ER levels are higher than 100 nM. The boundaries of the high agonism area can be clearly seen on the 2D projection of the graph (Figure 6B).

As follows from Figure 6B, similarly high levels of transcriptional activation can be achieved at significantly different levels of tamoxifen and ER. For instance, elevated gene expression is seen in the range of 1–50 nM of tamoxifen and 10–50 nM ER (area 2 on Figure 6B). However, a comparable agonistic effect can be also observed at lower and higher levels of tamoxifen and ER (areas 1 and 3). Another important direct consequence of the non-linear relationship between transcriptional activation and receptor level is that the same concentration of tamoxifen may cause significantly different effects depending on the background level of ER expression. Indeed, the model predicts that tamoxifen < 50 nM will have a stimulatory effect on ER-dependent transcription in cells with ER level lower than 80 nM, with anticipated maximal agonism for medium ER expression levels (10–30 nM ER, areas 1 and 2). However the same drug concentration will not cause any significant transcriptional response if the ER expression level is higher than 100 nM (area 4). Similarly, tamoxifen >100 nM will effectively suppress ER-induced transcription if ER expression is lower than 50 nM (area 5 on Figure 6B), but the same concentration of the drug may be inefficient and even cause a significant stimulation of gene expression at higher (>50 nM) levels of ER (area 3). This is in line with experimental findings, indicating that the development of tamoxifen resistance is often associated with elevated levels of estrogen receptor [54]. Importantly, as reported in [41], treatment of cancer cells with high concentration of tamoxifen can in itself stimulate ER expression, that potentially provides an additional basis for agonistic action of tamoxifen.

A similar trend of non-monotonic dependence of transcriptional activation on the amount of ER has also been predicted for lower levels of estradiol (Figure 6 C and D), though the effect was much less prominent than found for higher estradiol concentration. Interestingly, the model suggests that at a low level of background estradiol, a potentially high transcriptional response can be observed when both tamoxifen concentration and ER expression are sufficiently high (the area, highlighted by yellow and red on Figure 6C and D). In the range of low and medium tamoxifen concentrations, the magnitude of the transcriptional response is directly dependent on the background hormone concentration. (See Additional File 5 for an animation, showing how the characteristic "bump" in the region of medium ER and tamoxifen levels emerges and grows with a gradual increase in background estradiol concentration).

#### Experimental validation

To test our predictions about the non-monotonous dependence of ER -induced transcriptional activation on the level of ER expression, we analysed the stimulatory effects of estradiol and tamoxifen in several cell lines, which differed by their basal amounts of ER. We investigated four MCF-7 cell lines: wild type MCF-7, MCF-7/LCC1, MCF-7/LCC2 and MCF-7/LCC9 (Figure 7A). ER protein levels were significantly elevated in LCC1 and LCC2, as compared to wild type MCF-7 (from seven- to eleven-fold) and less markedly in LCC9 (Figure 7B). In each cell line we measured mRNA expression levels of four different estrogen-responsive genes, whose activity is known to be directly regulated by ER: trefoil factor 1 (TFF1), progesterone receptor (PGR), MYC and cathepsin D (CTSD). The measurements were run for a variety of conditions, including treatment of the cells with estradiol, tamoxifen and a combination of both.

Because of potential differences in transcriptional regulation of TFF1, PGR, MYC and CTSD genes their expression could not be possibly described with a single model with the same set of fixed parameters. For example, Won Jeong et al. [55] reported significant differences in co-regulator recruitment to target promoters of these four genes in breast cancer cells. Accounting for these dissimilarities in the model would require variation of parameters of ER binding with ERE and corresponding rate constants of transcriptional activation. Importantly, these parameters are not straightforward to estimate or measure. For that reason we were not seeking to directly quantitatively describe the experimental data in Figure 7 with our model. However, to allow for qualitative comparison of the model and experimental observations, we calculated the expression levels of a hypothetical ER-dependent gene for four different levels of ER expression (see Figure 7C).

As expected, there was no exact quantitative match between modelling results and the experimental data sets. However, a number of important qualitative trends observed experimentally were consistent with model predictions.

Namely, in agreement with the model, in the absence of any external ER agonists (control) all cells were capable of maintaining some basal level of gene expression, with the level of mRNA depending on ER in a non-monotonous way, noted for all four ER-dependent genes. Addition of tamoxifen alone resulted in some extra activation of transcription in the majority of genes (TFF1, PGR and CTD), with the exception of MYC in LCC9 and LCC1, where transcription dropped slightly, as compared to the control. Importantly, in line with the model predictions, the stimulatory effect of estradiol on transcriptional activation of all four genes was more prominent in MCF-7, LCC9 and LCC1 cells, than in LCC2 cells, which contained the highest amount of ER. Treatment of the cells with a combination of estradiol and tamoxifen resulted in a certain level of transcriptional inhibition as compared to estradiol alone. The only exception was the expression of PGR in LCC1 cells, where combining estrogen with tamoxifen led to some additional stimulation of transcription.

Thus, the experimental results were in qualitative agreement with the model predictions about non-monotonic dependence of ER-induced gene expression on the amount of ER. Indeed, the expression levels of each of the experimentally studied genes in most cases, and under a variety of treatment conditions, followed a trend of non-monotonous dependence on the ER level, with the maximal expression observed in either LCC9 or LCC1 cells, that are characterised by medium levels of ER expression. Cells with the highest amount of ER (LCC2) in most cases demonstrated a lower level of transcriptional activation as compared to LCC1 and LCC9.

Our model provides a consistent explanation for this non-intuitive phenomenon, shedding light on the potential mechanism underlying the detected differences in gene expression patterns between MCF-7, LCC9, LCC1 and LCC2 cell lines.

#### Combinatorial inhibition as the source of the observed non-monotonicity

We propose that the described phenomena of non-monotonic transcriptional response to the increasing concentration of ER fits well within the concept of "combinatorial inhibition" or "prozone effect" previously reported for other multi-component systems [56-58]. In summary, the concept suggests that certain types of multimeric protein complexes can be inhibited by high concentrations of one of its components. In 1997, Bray and Lay [58] used modelling approach to analyse 30 different oligomeric complexes and found that disproportionally high concentration of one of the complex components may cause suppression of multimeric complex assembly. This is due to accumulation of smaller incomplete complexes which sequester other proteins that are not present in high concentrations and thereby prevent the formation of fully functional complex. The effect was shown to be the most pronounced for those proteins which serve as the central core of the complex, forming multiple bonds with other complex components. These ideas were supported by a study of Levchenko et al. [57] who explored numerically the role of a scaffold protein in a MAPK pathway, and found that for any generic scaffold there exists a concentration value optimal for signal amplitude. These findings have been further confirmed experimentally by Chapman et al. [56], who studied the effect of over-expression of the scaffold protein Ste5 on the MAPK pathway in yeast and demonstrated that signal throughput exhibited a biphasic dependence on scaffold concentration.

In the system of ER-related signalling, ER is at the core of multimeric transcriptional complex, forming bonds with several ligands, ERE and multiple transcriptional co-regulators. Therefore, in line with predictions of [58] and with our own findings (Figures 6 and 7) an excess of ER is likely to cause a significant sequestration effect, resulting in the abundance of incomplete transcription complexes (like ER_2_D) and preventing from formation of both fully and partly functional complexes (like ER_2_H_2_D, ER_2_HD, ER_2_ID and ER_2_I_2_D).

Indeed, the analysis of the distribution of receptor-ligand complexes (Figure 6E) and the composition of transcription complexes present at ERE (Figure 6F) at various levels of ER expression reveals that at high levels of ER (e.g., as observed in LCC2), free ER_2_ dimers and ER_2_I complexes become the prevailing forms of the receptor-ligand complexes, whereas the relative amount of transcriptionally more active complexes (especially ER_2_H and ER_2_H_2_) significantly drops (Figure 6E). The excess of ER_2_ dimers as well as their higher affinity for ERE, as compared with that of ER_2_I complex (see Table 1), allows them to out-compete ER_2_I and other receptor-ligand complexes for binding with target promoters. Consequently, slow transcribing "incomplete" transcription complex (ER_2_D) become a prevailing type of complexes formed at ERE (Figure 6F), that causes a general drop in transcription at high expression level of ER, and may explain generally low expression level of ER-dependent genes in LCC2 cells (Figure 7A).

We suggest that the effect of such combinatorial inhibition may be an important factor in regulation of ER-induced transcription, both in the absence and in the presence of anti-estrogenic drugs.

### Tamoxifen agonism is dependent on the parameters associated with tamoxifen-receptor transcription complexes

As suggested by GSA, the ER-dependent protein expression is highly sensitive to the variation of the parameters, associated with the transcriptional activation induced by tamoxifen-receptor complexes (*k*_*sr,i*_, *K*_*19*_). Exploration of the steady state transcriptional response in the range of *k*_*sr,i*_ values (Figure 8A) revealed a potentially very strong transcriptional stimulation in the range of high dose tamoxifen at *k*_*sr,i*_ > 0.03. Interestingly, the model predicts that administration of the same concentration of tamoxifen can lead to either up- or down-regulation of transcription, depending on the value of *k*_*sr,i*_. For instance, for *k*_*sr,i*_ <0.02 high dose tamoxifen (>100 nM) would act as a pure antagonist, effectively suppressing ER-induced protein expression. However, in the range of *k*_*sr,i*_ >0.03 the same dose of the drug would result in significant transcriptional stimulation (area marked by yellow and red). Thus, even a slight variation in *k*_*sr,i*_ (e.g. caused by a change in intracellular microenvironment) may cause a significant change in transcriptional outcome, including a steep transition from clearly antagonistic action of tamoxifen to strong activatory effect.

Multiple recent studies have confirmed the crucial role of intra-cellular micro-environment in determining the ER-dependent transcriptional response. In particular, there is increasing evidence that the gene and cell-specific action of both estradiol and SERMs significantly depend on the presence of transcriptional co-activators and co-repressors, which, when recruited to the target promoter, are capable of promoting or hindering the activation of transcriptional machinery. For example, tamoxifen can act as ER antagonist in breast cancer cells [13], but, at the same time it can have a partial agonistic effect in endometrium, thus causing endometrial hyperplasia and even cancer [5]. These differences in tamoxifen action have been attributed to the different levels of transcriptional co-regulators present in breast and endometrium cells. Romano et al. [14] report that differential co-regulator recruitment explains the opposite transcriptional effect observed at a number of ER-regulated genes in response to OH-tamoxifen in breast cancer (T47D) and endometrial cancer (ECC1) cells. Moreover, they demonstrate that the transcriptional response to OH-tamoxifen in T47D and ECC1 cells can be, in fact, be inverted by over-expressing either co-activator SRC-1 or co-repressor SMRT.

Our model does not explicitly account for interaction of co-regulators with the transcription complex (partly because of the uncertainty of the kinetic parameters associated with these processes, that inevitably would over-complicate the model). However, the differential recruitment of co-regulators to the target promoters as well as their higher/lower expression levels can be imitated by assigning a higher or a lower value to corresponding transcription rate constants (e.g., *k*_*sr,i*_).

For instance, higher expression levels of co-activator SRC-1 found in endometrial cells [15] could be interpreted as a higher value of *k*_*sr,i*_ in our model. According to the graph in Figure 8A, this could result in high agonistic action of tamoxifen, given the dose of the drug is sufficiently high (>100 nM).

### Transcriptional response to tamoxifen rises along with the increase in the amount of ER- responsive genes

According to GSA predictions, the magnitude of transcriptional response to tamoxifen is strongly positively correlated with the number of ER-responsive genes (D_t_) (Figure 5). As seen from Figure 8B, in our model, the gradual increase in D_t_ resulted in significant elevation in the overall ER-induced protein expression in the wide range of tamoxifen concentrations, with more prominent effect observed in medium and low tamoxifen dose range (<100 nM).

One possible interpretation of this result is that overall ER-induced protein expression increases when more EREs become available for efficient ER binding.

This is in line with recent findings of Ross-Innes et al. [19] who studied ER-binding events in primary frozen breast cancer samples and demonstrated the acquisition of additional ER-binding regions in tumors with poor prognosis, as compared to good outcome samples. Such a modified ER binding profile in poor prognosis tumours was associated with upregulated expression of corresponding genes. Similar "reprogramming" of ER binding was also found in tamoxifen-resistant cancer cell line models. In terms of our model the acquisition of additional ER-binding regions can be interpreted as an efficient increase in D_t_. The model predicts (Figure 8B), that higher levels of D_t_ may be associated with the lack of tamoxifen efficiency. Indeed, at D_t_>0.3 nM even high concentrations of tamoxifen (I_t_> 1 μM) cause no more than 30% inhibition of ER-induced transcription. Moreover, in the dose range of 10–100 nM the drug may cause some additional transcriptional stimulation.

## Conclusions

The aim of this study was to develop a minimal mechanistic model of ER-dependent gene expression, which could be applied for the analysis of transcriptional response to natural hormone 17β-estradiol and SERMs in the broad range of their concentrations.

The developed model represents a system of algebraic equations, describing equilibrium binding of ER with ligands and DNA, linked to a simple ODE model of ER-induced gene expression. The model allows for the analysis of the effects of two types of ER ligands: natural hormone 17β-estradiol, and its external competitor tamoxifen. It should be noted that the model could be easily extended/modified to include other ligands or combination of those, including environmental phytoestrogens, if an appropriate data set for evaluation of relevant binding parameters becomes available.

The model was parameterised on several independent data sets, measured both *in vitro* and *in vivo* cell culture systems. Despite the broad diversity of experimental data used for calibration of its separate blocks, the resulting model was capable of satisfactorily describing the tamoxifen dose-dependence patterns of a reporter gene expression in HEK 293/hERα cell line, and provided a plausible explanation of the mechanisms underlying agonistic action of tamoxifen in the given cell line.

Apparently, many of the model parameters, including the total amounts of individual species and some of the binding parameters, are subject to biological variability and likely to be cell-type specific. Therefore, with a view to extending model conclusions to other cell types, we sought to explore the behaviour of the key model readout (steady state protein expression level, *p̄*) in the wider range of plausible parameter values, including those found in cancer cells. We used global sensitivity analysis to identify key control parameters, which are likely to have the biggest impact on the ER-dependent transcriptional response to tamoxifen. According to the GSA results, the most influential parameters were: the total amount of ER-responsive genes (D_t_), expression level of ER (ER_t_), background hormone concentration (H_t_) and parameters, associated with ER-tamoxifen transcription complex (k_sr,i_). Continuation of the steady state model solution within biologically plausible ranges of these control parameters revealed a number of interesting regulatory phenomena.

First of all, the transcriptional response to tamoxifen demonstrated explicit non-monotonous dependence on both tamoxifen dose and ER expression level, with distinct areas of high agonism within certain ranges of drug and ER concentrations (Figure 6). Model-based analysis of the composition of transcriptional response in the area of high agonism revealed that in the range of low and medium concentrations of tamoxifen, and at medium levels of ER expression, estradiol and tamoxifen may act synergistically, resulting in a significant elevation of ER-dependent transcription.

Importantly, the prominence of this effect was directly dependent on the background concentration of 17β-estradiol. At high dose tamoxifen the transcriptional response was found to be extremely sensitive to the parameters of transcription, induced by transcription complexes formed by ER bound with tamoxifen, that is in line with recent studies highlighting the important role of selective recruitment of transcriptional co-regulators by ER-tamoxifen complexes in defining agonistic action of tamoxifen [14,15].

Our findings suggest that transcriptional response to tamoxifen is a complex non-linear function of many variables, with the key control role belonging to ER expression level, hormone concentration, number of ER-responsive genes and the capacity of ER-tamoxifen complexes to stimulate transcription (e.g., by recruiting various co-regulators of transcription).

The majority of existing studies into the effects of SERMs and into associated risks of endometrial cancer have focused on the analysis of the impact of single individual factors on ER-dependent transcriptional activation and clinical outcome. For instance, association of higher estradiol concentration in serum with a higher risk of endometrial cancer has been reported [59]. There is also growing evidence of the important role of the specific changes in ER binding profile and transcriptional co-regulator recruitment in the emergence of tamoxifen resistance [7,19]. Other studies highlighted the role of ERα expression level in regulating ER-induced transcriptional response [54,60]. Our experiments presented in the current study provided evidence for non-linear dependence of ER-related transcriptional response on the level of ERα expression.

Our theoretical analysis suggests that the effect of each of these important factors on transcriptional outcome should not be considered in isolation, since it can be enhanced or attenuated by other key players implicated in controlling ER-mediated transcriptional response, whose role should not be overlooked.

At present, the only molecular biomarker routinely used in clinical practice for stratifying patients for therapy with tamoxifen is the presence of ERα expression in tumor cells. Any positive level of ERα expression is considered sufficient to justify the use of endocrine adjuvant therapy in almost all patients [61]. The lack of efficacy of tamoxifen therapy in significant group of patients as well as frequent emergence of drug resistance question the practical utility of single biomarker approach, and drive the research towards looking for more sophisticated tests, e.g., by including profiling for aberrations in additional genes, e.g., HER2, c-Myc, cyclin D [62-64]. Our analysis also suggests that more complex, or combinatorial, biomarkers are required. These could include more accurate quantitative assessment of individual ER expression level and concentration of estradiol, as well as profiling for key transcriptional co-regulators.

It must be emphasized, that our developed model is only a crude approximation of highly complicated ensemble of processes, associated with ER-mediated activation of transcriptional machinery. In current model implementation we have not explicitly included binding of ER with its transcriptional co-regulators (such as AIB1, SRC1, NCoR and SMART) and only indirectly accounted for activatory/inhibitory action of these molecules via variation of relevant rate constants of transcription. Because of these simplifications our theoretical conclusions, drawn from the model analysis, remain mainly of qualitative nature, and serve the purpose of attracting attention to potential non-linear effects in the system of ER-induced transcription and laying the basis for their further investigation. Ideally, for more reliable approximation of the effects of SERMs in cancer cell lines as well as for predicting potential outcome of tamoxifen therapy in patients, further model refinement and more systematic experimental studies are required, which would allow more accurate mapping of the "high agonism" area in the multi-dimensional space of the key biological parameters, controlling ER-dependent gene expression.

## Methods

### Equilibrium Model of ER dimerisation, binding with ligands and ERE DNA

For the processes 1–20, depicted in Figure 1A and 1B, the dissociation constants are defined as follows:

$$\begin{array}{l} K_{1}=\frac{ER^{2}}{ER_{2}};K_{2}=\frac{ER_{2}\cdot H}{ER_{2}H};K_{3}=\frac{ER_{2}H\cdot H}{ER_{2}H_{2}};K_{4}=\frac{ER\cdot H}{ERH};\\ K_{5}=\frac{ER\cdot ERH}{ER_{2}H};K_{6}=\frac{ERH^{2}}{ER_{2}H_{2}};K_{7}=\frac{ER_{2}\cdot I}{ER_{2}I};K_{8}=\frac{ER_{2}I\cdot I}{ER_{2}I_{2}};\\ K_{9}=\frac{ER\cdot I}{ERI};K_{10}=\frac{ER\cdot ERI}{ER_{2}I};K_{11}=\frac{ERI^{2}}{ER_{2}I_{2}};\\ K_{12}=\frac{ERI\cdot ERH}{ER_{2}HI};K_{13}=\frac{ER_{2}I\cdot H}{ER_{2}HI};K_{14}=\frac{ER_{2}H\cdot I}{ER_{2}HI}\\ K_{15}=\frac{ER_{2}\cdot D}{ER_{2}D};K_{16}=\frac{ER_{2}H\cdot D}{ER_{2}HD};K_{17}=\frac{ER_{2}H_{2}\cdot D}{ER_{2}H_{2}D};\\ K_{18}=\frac{ER_{2}I\cdot D}{ER_{2}ID}K_{19}=\frac{ER_{2}I_{2}\cdot D}{ER_{2}I_{2}D};K_{20}=\frac{ER_{2}HI\cdot D}{ER_{2}HID}\text{;} \end{array}$$

Under equilibrium conditions the following detailed balances exist in the system:

$$K_{1}K_{2}=K_{4}K_{5}$$

$$K_{1}K_{2}K_{3}=K_{4}^{2}K_{6}$$

$$K_{1}K_{7}=K_{9}K_{10}$$

$$K_{1}K_{7}K_{8}=K_{9}^{2}K_{11}$$

$$K_{2}K_{14}=K_{7}K_{13}$$

$$K_{9}K_{12}=K_{5}K_{14}$$

This means that six out of 14 K_d_s depicted in Figure 1A depend on others and can be expressed as follows:

$$\begin{array}{l} K_{5}=\frac{K_{1}K_{2}}{K_{4}};K_{6}=\frac{K_{1}K_{2}K_{3}}{K_{4}^{2}};K_{10}=\frac{K_{1}K_{7}}{K_{9}};K_{11}=\frac{K_{1}K_{7}K_{8}}{K_{9}^{2}};\\ K_{12}=\frac{K_{1}K_{7}K_{13}}{K_{4}K_{9}};K_{14}=\frac{K_{7}K_{13}}{K_{2}}\text{.} \end{array}$$

Thus, under equilibrium conditions, the distribution of species in the system is defined by the following system of algebraic equations:

For ER binding with the hormone:

(1) $$ER_{2}=\frac{ER^{2}}{K_{1}}$$

(2) $$ER_{2}H=\frac{ER^{2}}{K_{1}}\frac{H}{K_{2}}$$

(3) $$ER_{2}H_{2}=\frac{ER^{2}}{K_{1}}\frac{H^{2}}{K_{2}K_{3}}$$

(4) $$ERH=\frac{ER\cdot H}{K_{4}}$$

For ER binding with the inhibitor:

(5) $$ER_{2}I=\frac{ER^{2}}{K_{1}}\frac{I}{K_{7}}$$

(6) $$ER_{2}I_{2}=\frac{ER^{2}}{K_{1}}\frac{I^{2}}{K_{7}K_{8}}$$

(7) $$ERI=\frac{ER\cdot I}{K_{9}}$$

(8) $$ER_{2}HI=\frac{ER^{2}}{K_{1}}\frac{I\cdot H}{K_{7}K_{13}}$$

For binding of ER dimer complexes with ERE DNA (D):

(9) $$ER_{2}D=\frac{ER^{2}}{K_{1}}\frac{D}{K_{15}}$$

(10) $$ER_{2}HD=\frac{ER^{2}}{K_{1}}\frac{H}{K_{2}}\frac{D}{K_{16}}$$

(11) $$ER_{2}H_{2}D=\frac{ER^{2}}{K_{1}}\frac{H^{2}}{K_{2}K_{3}}\frac{D}{K_{17}}$$

(12) $$ER_{2}ID=\frac{ER^{2}}{K_{1}}\frac{I}{K_{7}}\frac{D}{K_{18}}$$

(13) $$ER_{2}I_{2}D=\frac{ER^{2}}{K_{1}}\frac{I^{2}}{K_{7}K_{8}}\frac{D}{K_{19}}$$

(14) $$ER_{2}HID=\frac{ER^{2}}{K_{1}}\frac{I\cdot H}{K_{7}K_{13}}\frac{D}{K_{20}}$$

For simplicity we assume that in the system shown in Figure 1 the total concentrations of estrogen receptor (ER_t_), hormone (H_t_), inhibitor (I_t_) and DNA (D_t_) are conserved. This assumption is justified for the model describing the data obtained in *in vitro* studies on purified receptor and ERE, where the total concentration of each of the species is unambiguously defined by the experimental protocol. The mass conservation approximation also holds for *in vivo* cellular systems, assuming that cellular amounts of ER and DNA are maintained at some quasi-steady state level, resulting from the balance between the processes of their synthesis and degradation, whereas hormone and drug concentrations are controlled experimentally.

This allows definition of the concentrations of free forms of these species as follows:

(15) $$\begin{array}{l} ER=ER_{t}-ERH-ERI-2\cdot (ER_{2}+ER_{2}H+ER_{2}H_{2}+ER_{2}I+ER_{2}I_{2}+ER_{2}HI+\\ ER_{2}D+ER_{2}HD+ER_{2}H_{2}D+ER_{2}ID+ER_{2}I_{2}D+ER_{2}HID) \end{array}$$

(16) $$\begin{array}{l} H=H_{t}-ERH-ER_{2}H-ER_{2}HI-ER_{2}HID-ER_{2}HD\\ -2\cdot (ER_{2}H_{2}+ER_{2}H_{2}D) \end{array}$$

(17) $$I=I_{t}-ERI-ER_{2}I-ER_{2}HI-ER_{2}HID-ER_{2}ID-2\cdot \left(ER_{2}I_{2}+ER_{2}I_{2}D\right)$$

(18) $$D=D_{t}-ER_{2}D-ER_{2}HD-ER_{2}HID-ER_{2}ID-ER_{2}H_{2}D-ER_{2}I_{2}D$$

### Simplified model of ER -dependent transcription/translation

To describe the ER-dependent expression of an estrogen-responsive gene, we linked the algebraic system (1–18) to a simplified kinetic model of transcription and translation of an ER-dependent gene, describing synthesis and degradation of corresponding mRNA (*r*) and protein (*p*):

(19) $$\begin{array}{l} \frac{dr}{dt}=k_{\mathrm{sr}}(ER_{2}HD+ER_{2}H_{2}D)+k_{sr,i}(ER_{2}ID+ER_{2}I_{2}D)+\\ +k_{sr,hi}ER_{2}HID+k_{sr,b}ER_{2}D-k_{\mathrm{dr}}r\text{;} \end{array}$$

(20) $$\frac{dp}{dt}=k_{\mathrm{sp}}r-k_{\mathrm{dp}}p$$

Here we assume that the rate of mRNA synthesis is proportional to the concentration of transcription complexes assembled at ERE, and that different forms of ER_2_-ligand complexes differ by their capability to induce transcription. In particular, we assume that unliganded ER dimer can stimulate transcription at some basal level with the rate constant k_sr,b_; complexes ER_2_H and ER_2_H_2_ have an equal potency to induce transcription (k_sr_); similarly, ER_2_I and ER_2_I_2_ stimulate transcription with the rate constant k_sr,i_, whereas the heterodimer ER_2_HI has a different capacity to induce transcription (k_sr,hi_). Those assumptions were based on current understanding of the potency of various forms of ER to induce transcription. For example, Fowler et al. [24,25] demonstrated that increased ERα expression in MCF7 cells resulted in activation of ER-responsive gene even in the absence of estrogen, that proves the ability of unliganded ER dimers to bind to ERE and stimulate transcription. The ability of tamoxifen to induce transcription has been confirmed in multiple studies on agonist/antagonist effects of SERMs [11,38,41,42,65].

It is easy to demonstrate that in the system (19–20) the steady state concentrations of RNA (*rˉ *) and protein (*pˉ *) are fully defined by the concentrations of active transcription complexes as follows:

(21) $$\overset{Â¯}{r}=\frac{v_{\mathrm{tr}}}{k_{\mathrm{dr}}}\text{;}$$

(22) $$\overset{Â¯}{p}=\frac{v_{\mathrm{tr}}}{\kappa }\text{;}$$

where

(23) $$v_{\mathrm{tr}}=k_{\mathrm{sr}}\left(ER_{2}HD+ER_{2}H_{2}D\right)+k_{sr,i}\left(ER_{2}ID+ER_{2}I_{2}D\right)+k_{sr,hi}ER_{2}HID+k_{sr,b}ER_{2}D\text{;}$$

(24) $$\kappa =\frac{k_{\mathrm{dp}}k_{\mathrm{dr}}}{k_{\mathrm{sp}}}$$

Here *v*_*tr*_ describes the overall rate of ER-dependent transcription, stimulated by various types of transcription complexes; κ is the ratio of the rate constants of protein degradation, mRNA degradation and protein synthesis.

Thus, our simplified model of ER-dependent gene expression represents a system of algebro-differential equations (1–20). The steady state solution of the system can be found by solving the system of algebraic equations (1–18, 21–24). Importantly, using an equilibrium and steady state approximation allowed us to reduce the number of independent model parameters from 45 to 23.

### Global sensitivity analysis (GSA)

GSA of the model of ER-dependent gene expression was performed according to the following algorithm:

### Step1: Definition of the set of parameters to perturb and setting their boundaries

We perturbed all independent kinetic parameters of the model (dissociation and reaction rate constants) and total concentrations of model species, with the exception of the inhibitor concentration (I_t_), whose value was fixed to a certain value during each run of GSA. In total we performed 138 GSA runs for 138 different concentrations of tamoxifen to evenly cover the drug dose range of 0.01-3000 nM on a logarithmic scale. The full list of parameters used for GSA and their constraints can be found in Additional File 6.

### Step 2: Sampling N random parameter sets from the parameter space

To randomly sample the parameter sets from the hypercube defined by parameter ranges we used Latin Hypercube Sampling (LHS) algorithm [66]. LHS represents a variant of stratified sampling based on simultaneous variation of all input parameters, that ensures that individual parameter ranges are evenly covered, and each parameter combination is unique. For our analysis we sampled 50000 random parameter sets.

### Step 3: Simulating the model for each parameter set

For each randomly selected parameter set we ran a simulation of the model and calculated the steady state level of protein expression *pˉ * as defined in equations (22–24).

### Step 4. Calculating sensitivity indices

To analyse the sensitivity of the steady state protein expression *pˉ * to the variation of model parameters we used a variant of Partial Rank Correlation Coefficient (PRCC) analysis, as one of the most efficient and reliable sampling-based techniques [67]. For each fixed dose of tamoxifen, for each model parameter *K*_*j*_, we calculated the PRCC index, that represents a standardized sensitivity metric of correlation between the value of the model readout (*pˉ *) and model parameter *K*_*j*_ .To reduce the influence of nonlinearity, the correlation was calculated based upon ranks rather than absolute values.

PRCC between *K*_*j*_ and *pˉ * was calculated as the correlation coefficient *r*_*j*_ between the two residuals *k*_*j*_ = *K*_*j*_^*R*^ − *K*_*j*_^*L*^ and *p* = *p*^*R*^ − *p*^*L*^, where *K*_*j*_^*R*^ and *p*^*R*^ are rank transformed *K*_*j*_ and *pˉ *; *K*_*j*_^*L*^ and *p*^*L*^ are the linear regression models defined as follows [67]:

$$\begin{array}{c} K_{j}^{L}=a_{0}+\overset{n}{\underset{\begin{array}{c} l=1\\ l\neq j \end{array}}{\sum }}a_{l}K^{R}{}_{l};p^{L}\\ =b_{0}+\overset{n}{\underset{\begin{array}{c} l=1\\ l\neq j \end{array}}{\sum }}b_{l}K_{l}^{R} \end{array}$$

Thus

$$r_{j}=\frac{\sum_{i=1}^{N}\left(k_{\mathrm{ij}}-\mu_{k}\right)\left(p_{i}-\mu_{p}\right)}{\sqrt{\sum_{i=1}^{N}{\left(k_{\mathrm{ij}}-\mu_{k}\right)}^{2}\sum_{i=1}^{N}{\left(p_{i}-\mu_{p}\right)}^{2}}}\text{,}$$

where N is the number of parameter sets sampled from the model parameter space; *μ*_*k*_ and *μ*_*p*_ are respective sample means.

Importantly, the sign of a PRCC indicates how the variation of each parameter affects the value of *pˉ *: the positive index corresponds to the parameter whose higher value is likely to be associated with a higher transcriptional response, and vice versa. The value of PRCC indices are distributed between - 1 and 1 with 0 indicating those parameters to whose variation the model output is completely insensitive.

### Computation

Model construction and simulation was performed with the use of the DBSolve package [68]. Fitting the model to experimental data involved minimisation of least square deviation between experimental and theoretical curves, with the use of Hooke and Jeeves algorithm as implemented in DBsolve package. Global sensitivity analysis made use of KINSOL solver of SUNDIALS [69].

### Experimental methods

#### Cell culture

MCF-7 cells were routinely grown in phenol red containing Dulbecco’s Modified Eagle medium (DMEM) supplemented with 10% fetal calf serum (FCS), Penicillin (100 Units/ml) and Streptomycin (100 mg/ml). LCC1, LCC2 and LCC9 cells (source: Dr.Robert Clarke, V.T.Lombardi Cancer Research Center, Georgetown University Medical School, Washington, D.C., USA) were routinely kept in phenol red free containing Dulbecco’s Modified Eagle medium (DMEM) supplemented with 5% dextran activated charcoal stripped fetal calf serum (DCC), Penicillin (100 Units/ml) Streptomycin (100 mg/ml) and 2mM Glutamine [70-72] All cells were grown at 37°C in 5% CO_2_. To determine the effects of 17β-estradiol (E_2_) and tamoxifen on mRNA expression, MCF-7 cells were seeded in 6 well plates in phenol red containing DMEM with 10%FBS for 24 h. The media was changed to phenol-red-free DMEM with 5% DCC for 48 h. The cells were then supplemented with media containing either 1 nM E_2_, 1 μM tamoxifen or both. LCC1, LCC2 and LCC9 cells were seeded in 6 well plates in phenol-red-free containing DMEM with 5%DCC and after 24h supplemented with E_2_ and/or tamoxifen.

#### RNA extraction and RT-PCR

Extraction of total RNA from whole cells was performed using Tri-Reagent (Sigma, Poole, Dorset) as per manufacturer instructions. RNA concentration was measured using a spectrophotometer. QuantiTect™SYBR®Green system (Quiagen cat#204243) was used according to the manufacturers instructions for one step RT-PCR in a total of 15μl reaction volumes including 0.5 μM each primer and 40ng RNA. Real Time cycler conditions were RT: 50°C for 30 min; PCR: initial activation 95°C for 15 min; followed by 40 cycles of denaturation 94°C for 15 sec, annealing 57°C for 30 sec, extension 72°C for 30 sec; and a final extension of 72°C for 60 sec. The following primers were used:

TFF1: TTGTGGTTTTCCTGGTGTCA and CCGAGCTCTGGGACTAATCA

PGR: GTCAGTGGGCAGATGCTGTA and AGCCCTTCCAAAGGAATT

CTSD: CCCGAGGTGCTCAAGAACTA and TCACGTAGGTGCTGGACTTG

MYC: TTCGGGTAGTGGAAAACCAG and AGCAGCTCGAATTTCTTCCA

ACTIN: CTACGTCGCCCTGGACTTCGAGC and GATGGAGCCGCCGATCCACACGG

cDNA was analyzed using a Rotorgene 2000 (Corbett Research, Cambridge, UK).

## Abbreviations

ER: Estrogen receptor α; ERE: Estrogen response element; SERM: Selective estrogen receptor modulator; GSA: Global sensitivity analysis.

## Competing interests

The authors declare that they have no competing interests.

## Authors’ contributions

GL designed the study, constructed the model, run simulations and parameter fitting, analysed literature data and GSA results, and wrote the manuscript; AY ran global sensitivity analysis of the model and generated 3D graphs; KM performed the experiments; SPL and DJH conceived the idea of the study, helped to identify suitable literature and data, designed the experiments, contributed to analysis of results and critically revised the manuscript.

## Supplementary Material

Additional file 1 Full ODE model of ER-induced gene expression. Click here for file

Additional file 2 Parameterisation of the separate blocks of the model. Click here for file

Additional file 3: Figure S1 The full spectrum of sensitivity of ER-dependent protein expression to the variation of the model parameters, calculated in the broad range of tamoxifen concentrations. Click here for file

Additional file 4 Estimation of biologically plausible parameter constraints. Click here for file

Additional file 5 **An animation, demonstrating how the 3D graph shown in Figure **6**C evolves with gradual increase in background estradiol concentration.** Click here for file

Additional file 6 The list of parameters and parameter constraints for GSA Click here for file
