# Supplementary material for: A model of estrogen-related gene expression reveals non-linear effects in transcriptional response to tamoxifen
Source: BMC Syst Biol. 2012 Nov 8;6:138. doi: 10.1186/1752-0509-6-138 (PMC3573949; doi:10.1186/1752-0509-6-138)
Supplement: Additional file 1 — Full ODE model of ER-induced gene expression. [file 1752-0509-6-138-S1.pdf]

Additional File 1.

Full ODE model of ER-induced gene expression.

The reaction rates for each of the processes depicted in Figure 1 of the manuscript were formulated as follows:

$$v_1 = k_1([ER]^2 - K_1[ER_2]);$$
$$v_2 = k_2([ER_2][H] - K_2[ER_2H]);$$
$$v_3 = k_3([ER_2H][H] - K_3[ER_2H_2]);$$
$$v_4 = k_4([ER][H] - K_4[ERH]);$$
$$v_5 = k_5([ER][ERH] - K_1K_2[ER_2H]/K_4);$$
$$v_6 = k_6([ERH]^2 - K_1K_2K_3[ER_2H_2]/K_4^2);$$
$$v_7 = k_7([ER_2][I] - K_7[ER_2I]);$$
$$v_8 = k_8([ER_2I][I] - K_8[ER_2I_2]);$$
$$v_9 = k_9([ER][I] - K_9[ERI]);$$
$$v_{10} = k_{10}([ER][ERI] - K_1K_7[ER_2I]/K_9);$$
$$v_{11} = k_{11}([ERI]^2 - K_1K_7K_8[ER_2I_2]/K_9^2);$$
$$v_{12} = k_{12}([ERI][ERH] - K_1K_7K_{13}[ER_2HI]/(K_4K_9));$$
$$v_{13} = k_{13}([ER_2I][H] - K_{13}[ER_2HI]);$$
$$v_{14} = k_{14}([ER_2H][I] - K_7K_{13}[ER_2HI]/K_2);$$
$$v_{15} = k_{15}([ER_2][D] - K_{15}[ER_2D]);$$
$$v_{16} = k_{16}([ER_2H][D] - K_{16}[ER_2HD]);$$
$$v_{17} = k_{17}([ER_2H_2][D] - K_{17}[ER_2H_2D]);$$
$$v_{18} = k_{18}([ER_2][D] - K_{18}[ER_2ID]);$$
$$v_{19} = k_{19}([ER_2I_2][D] - K_{19}[ER_2I_2D]);$$
$$v_{20} = k_{20}([ER_2HI][D] - K_{20}[ER_2HID]);$$
$$v_{21} = k_{sr,b}[ER_2D] + k_{sr,H}([ER_2HD] + [ER_2H_2D]) + k_{sr,I}([ER_2ID] + [ER_2I_2D]) + k_{sr,HI}[ER_2HID];$$
$$v_{22} = k_{dr}r;$$
$$v_{23} = k_{sp}r;$$
$$v_{24} = k_{dp}p;$$

where k_i are reaction rate constants, K_i - dissociation constants, and [species] correspond to the concentration of the receptor-ligand complexes and other species, depicted in Fig.1.

The evolution of the molecular species in time is described by the following ODE system and four algebraic equations, formulated based on mass conservation laws for total concentration of the hormone (H_t), receptor (ER_t), inhibitor (I_t) and DNA ERE (D_t):

$$\frac{d[ER]}{dt} = -2v_1 - v_4 - v_5 - v_9 - v_{10};$$

$$\frac{d[ER_2H]}{dt} = v_2 + v_5 - v_3 - v_{14} - v_{16};$$

$$\frac{d[ERH]}{dt} = v_4 - v_5 - 2v_6 - v_{12};$$

$$\frac{d[ER_2I]}{dt} = v_7 + v_{10} - v_8 - v_{13} - v_{18};$$

$$\frac{d[ERI]}{dt} = v_9 - v_{10} - 2v_{11} - v_{12};$$

$$\frac{d[ER_2HI]}{dt} = v_{12} + v_{13} + v_{14} - v_{20};$$

$$\frac{d[H]}{dt} = -v_2 - v_3 - v_4 - v_{13};$$

$$\frac{d[I]}{dt} = -v_7 - v_8 - v_9 - v_{14};$$

$$\frac{d[D]}{dt} = -v_{15} - v_{16} - v_{17} - v_{18} - v_{19} - v_{20};$$

$$\frac{d[ER_2HD]}{dt} = v_{16};$$

$$\frac{d[ER_2HID]}{dt} = v_{20};$$

$$\frac{d[ER_2ID]}{dt} = v_{18};$$

$$\frac{d[ER_2I_2D]}{dt} = v_{19};$$

$$\frac{d[ER_2H_2D]}{dt} = v_{17};$$

$$\frac{d[r]}{dt} = v_{21} - v_{22};$$

$$\frac{d[p]}{dt} = v_{23} - v_{24};$$

$$[ER_2D] = D_t - [D] - [ER_2I_2D] - [ER_2ID] - [ER_2H_2D] - [ER_2HD] - [ER_2HID];$$

$$[ER_2H_2] = 0.5(H_t - [H] - [ERH] - [ER_2H] - [ER_2HI] - [ER_2HID] - [ER_2HD]) - [ER_2H_2D];$$

$$[ER_2I_2] = 0.5(I_t - [I] - [ERI] - [ER_2I] - [ER_2HI] - [ER_2HID] - [ER_2ID]) - [ER_2I_2D];$$

$$[ER_2] = 0.5(ER_t - [ER] - [ERH] - [ERI]) - [ER_2HI] - [ER_2H_2] - [ER_2I_2] - [ER_2H] - [ER_2I] -$$
$$- [ER_2H_2D] - [ER_2I_2D] - [ER_2HD] - [ER_2ID] - [ER_2HID] - [ER_2D];$$

The resulting model has 45 independent parameters (41 kinetic constants and 4 total concentrations of species)
