# Supplementary material for: A model of estrogen-related gene expression reveals non-linear effects in transcriptional response to tamoxifen
Source: BMC Syst Biol. 2012 Nov 8;6:138. doi: 10.1186/1752-0509-6-138 (PMC3573949; doi:10.1186/1752-0509-6-138)
Supplement: Additional file 2 — Parameterisation of the separate blocks of the model. [file 1752-0509-6-138-S2.pdf]

Additional File 2.

Calibration of the separate blocks of the model of ER-induced gene expression

1. Interaction of ER with ligands.

1.1. Interaction of ER with its natural ligand - hormone 17β-estradiol.

To identify parameters of ER binding with the hormone we used two types of data:
1) the data on equilibrium binding of purified recombinant hERα with 17β-estradiol, measured at four different concentrations of hERα (Obourn *et al*, 1993);
2) the data on equilibrium competitive binding of 17β-estradiol with hERα, measured at the presence of fixed concentration of $[^3H]$17β-estradiol (Bolger *et al*, 1998).
To fit the data published in (Obourn *et al*, 1993), we used the submodel, consisting of equations (1-4) and (15-16). To describe the data obtained in (Bolger *et al*, 1998) a larger submodel was used, consisting of equations (1-8) and (15-17), where $[^3H]$17β-estradiol was assigned a role of an inhibitor I. From fitting the model against these data sets we managed to evaluate four independent parameters of the system - K1, K2, K3 and K4; the constants K5 and K6 depend on K1, K2, K3 and K4 and were calculated as explained in model description. The identified parameter values are given in Table 1.

1.2. ER binding with Tamoxifen

To evaluate dissociation constants for reactions involving tamoxifen (K7, K8, K9, K13) we used the data on equilibrium competitive binding of tamoxifen with hERα, measured in the presence of fixed concentration of $[^3H]$17β-estradiol (Bolger *et al*, 1998). To fit the data we used a submodel, consisting of equations (1-8) and (15-17), where tamoxifen was assigned a role of an inhibitor I. The dissociation constants for other complexes involving tamoxifen (K10, K11, K12, K14) were dependent on K7, K8, K9 and K13, and calculated as described in the main text. See Table 1 and Figure 2 for fitting results.

2. Binding of ER_2-ligand complexes with DNA

At the next step we evaluated the parameters of the reactions, describing the interaction of ER dimer complexes with DNA ERE. As an initial approximation of these parameters we used literature available estimates (see Table 1), obtained in experimental systems with a perfectly palindromic ERE sequence derived from the vitellogenin A2 gene (VitERE). It is worth noting that in most cases is'nt possible to experimentally discriminate between binding of ER_2H and ER_2H_2 to ERE. Since the majority of existing studies have used high hormone concentrations (5-10 molar excess with regard to ER), we initially attributed the available estimates to binding of ER_2H_2 to ERE. However these values then have been re-adjusted, to provide the best match to the data on 17β-estradiol and Tamoxifen agonism effecst in the HEK 293/hERα reporter cell line (Barkhem *et al*, 1998), see Figure 2D.

3. ER-dependent gene expression.

Finally, the parameters of the ODE system describing the processes of transcription and translation, have been evaluated.
There are no existing estimates for the reaction rate constants of transcriptional activation caused by different forms ER-related transcription complexes. Therefore for these constants we first used biologically plausible approximations, and then refined them via fitting the model against experimental data on the effects of estradiol and tamoxifen on the ER-responsive gene expression. Initial approximations of the constants were based on the following assumptions: the ER dimers bound with the hormone (ER_2H_2 and ER_2H) have the highest capacity to activate ER-dependent transcription, resulting in the effective transcription rate of one mRNA copy per every 5 seconds ($k_{sr,h} = 0.2\ s^{-1}$). This value was based on the estimate for the maximal transcription rate of one mRNA molecule per every 5-8 s (Iyer and Struhl, 1996; Struhl, 2007)). All other receptor dimers, including those bound to the inhibitor, were assumed to be also capable of inducing transcription, but with a lower capacity. We assumed that $k_{sr,\ h} > k_{sr,\ hi} > k_{sr,i} > k_{sr,b}$. For example, the receptor dimers, bound with tamoxifen, were assumed to stimulate transcription 5-10-fold less efficiently than 17β-estradiol- bound dimers.
The final values of these rate constants (see Table 2) were estimated via fitting the steady state solution of the full model against the data on 17β-estradiol and tamoxifen effect on the expression of an ER-dependent reporter gene pΔERE2-ALP , encoding human placental alkaline phosphatase (ALP) in the human 293 kidney epithelial cell line, containing hERα reporter vector (HEK 293/ hERα) (Barkhem *et al*, 1998). Note, that the use of dose dependence data for fitting the model parameters prevented independent evaluation of the rates constants k_{dr}, k_{sp} and k_{dp}, but allowed estimation of their ratio κ, as defined in the equation (22).

References

Barkhem T, Carlsson B, Nilsson Y, Enmark E, Gustafsson J, Nilsson S (1998) Differential response of estrogen receptor alpha and estrogen receptor beta to partial estrogen agonists/antagonists. *Mol Pharmacol* **54:** 105-112.

Bolger R, Wiese TE, Ervin K, Nestich S, Checovich W (1998) Rapid screening of environmental chemicals for estrogen receptor binding capacity. *Environ Health Perspect* **106:** 551-557.

Iyer V, Struhl K (1996) Absolute mRNA levels and transcriptional initiation rates in Saccharomyces cerevisiae. *Proc Natl Acad Sci U S A* **93:** 5208-5212.

Obourn JD, Koszewski NJ, Notides AC (1993) Hormone- and DNA-binding mechanisms of the recombinant human estrogen receptor. *Biochemistry* **32:** 6229-6236.

Struhl K (2007) Transcriptional noise and the fidelity of initiation by RNA polymerase II. *Nat Struct Mol Biol* **14:** 103-105.
