# Supplementary material for: A model of estrogen-related gene expression reveals non-linear effects in transcriptional response to tamoxifen
Source: BMC Syst Biol. 2012 Nov 8;6:138. doi: 10.1186/1752-0509-6-138 (PMC3573949; doi:10.1186/1752-0509-6-138)
Supplement: Additional file 3: Figure S1 — The full spectrum of sensitivity of ER-dependent protein expression to the variation of the model parameters, calculated in the broad range of tamoxifen concentrations. [file 1752-0509-6-138-S3.pdf]

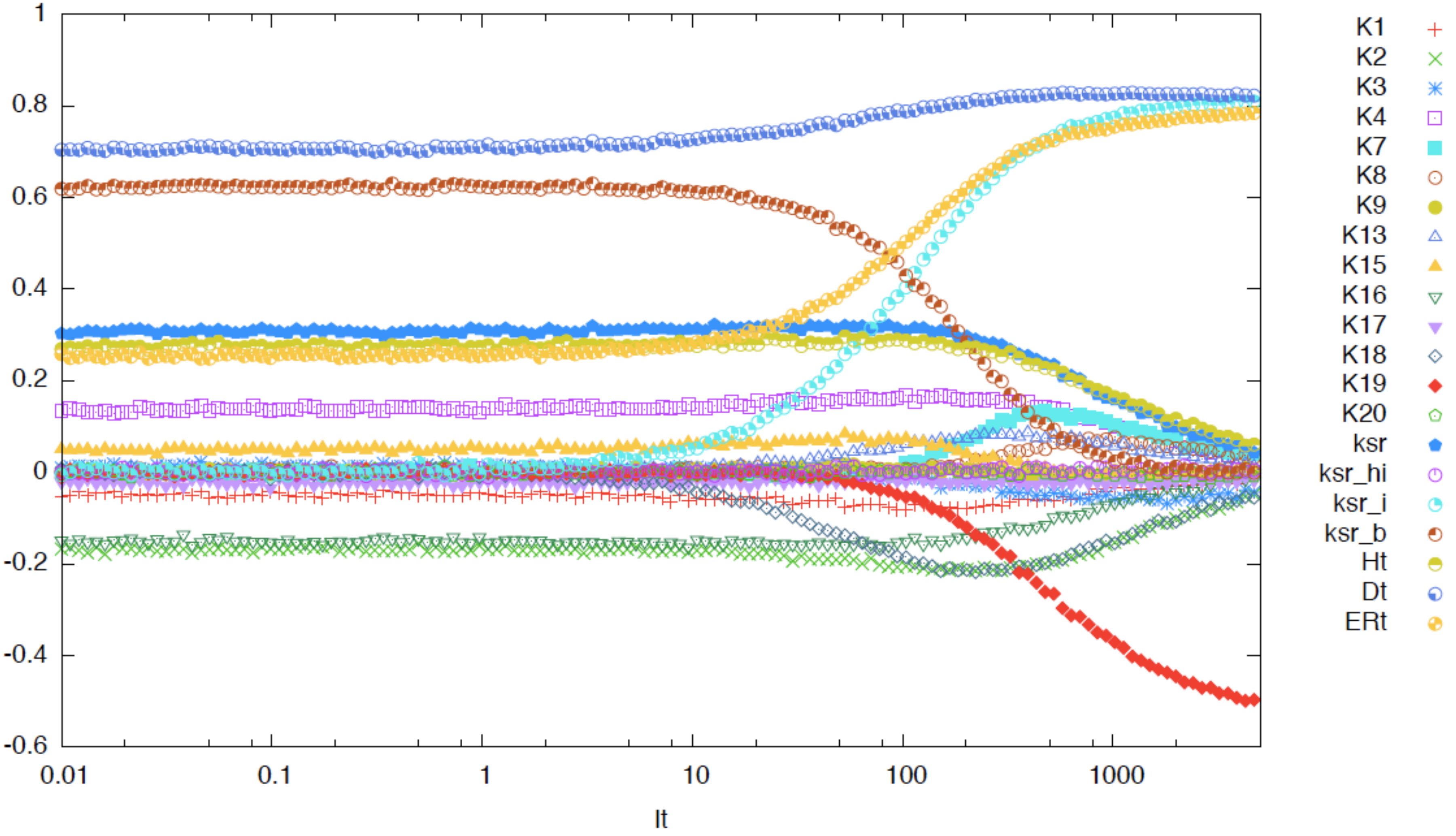

Supplementary Figure S1. The full spectrum of sensitivity of ER-dependent protein expression $\overline{p}$ to the variation of the model parameters, calculated in the broad range of tamoxifen concentrations. Each colored label on the graph represents a value of PRCC coefficient between $\overline{p}$ and corresponding parameter, calculated at certain fixed tamoxifen concentration It, as shown on the x axis.
