# Supplementary material for: A model of estrogen-related gene expression reveals non-linear effects in transcriptional response to tamoxifen
Source: BMC Syst Biol. 2012 Nov 8;6:138. doi: 10.1186/1752-0509-6-138 (PMC3573949; doi:10.1186/1752-0509-6-138)
Supplement: Additional file 4 — Estimation of biologically plausible parameter constraints. [file 1752-0509-6-138-S4.pdf]

Additional File 4

Estimation of biologically plausible ranges for ER, ERE, Ht and It.

17β-estradiol (H_t)

Biological variation of estradiol levels was estimated from recent studies (Lonning *et al*, 2011) measuring plasma, benign breast and breast cancer estrogen levels in pre- and postmenopausal women.

In postmenopausal women the plasma levels of estradiol are in the range of 15-25 pM, whereas premenopausal levels are around 0.2 nM. The concentration of estradiol in benign breast tissue is only slightly higher (< 2-fold) than in plasma in both pre and postmenopausal groups. However ER positive tumors have significantly higher levels of estadiol (0.14-0.5 nM in postmenopausal and 0.5-3 nM in premenopausal women, that is 9-fold and 4-fold higher than in benign tissue). These estimates were derived from data presented in (Lonning *et al*, 2011) under assumption that 100 fmol/g tissue roughly corresponds to 100 pmol/L.

Estrogen receptor (ER_t)

There are several estimates available for MCF-7 cells. Basing on the available data on the amount of anti-estrogen binding sites, (Olea-Serrano *et al*, 1985; Reddel *et al*, 1985)) and assuming that average size of MCF-7 cell is roughly 25 μm in diameter (Vona *et al*, 2000), the wild type MCF-7 cells may contain 10-50 nM ERα; The estimate obtained in (Murdoch and Gorski, 1991) is also in the range of 10-30 nM. Importantly, ERα expression levels are likely to be subject to broad biological variability. For example, (Punnonen *et al*, 1984) report cytoplasmic estrogen receptor concentrations in the endometrium of Finnish and Japanese premenopausal women as 246.9 +/- 46.2 and 45.7 +/- 17.1 fmol/mg protein respectively. Assuming that 1 fmol/mg protein corresponds to about 100 fmol/g wet weight of tissue or 100 pmol/L this roughly gives the concentration of 25 nM ER for Finnish and 5 nM ER for Japanese women. Presumably, in ER positive cancerous cells these values can be significantly higher.

Thus, in current study we explored the variation of ER expression level in a broad range to allow studying the potential effect of both extremely low and significantly elevated levels of ER: ERt value was varied from 1pm (sensitivity cut-off for ER negative cells) to up to 300 nM in ER positive cells.

Tamoxifen (I_t)

The range of plausible tamoxifen concentrations was estimated basing on the data of Kisanga et al (Kisanga *et al*, 2004), who measured tamoxifen concentrations in serum and breast cancer tissue during three dose regimens in a randomized preoperative trial. In this study, the average tamoxifen concentration in blood plasma was 10-20 nM at low dose (1 mg daily), and 200 nM at high dose tamoxifen (20 mg daily). Importantly, in cancer tissue tamoxifen content tends to be significantly higher than in plasma and its average concentration ranges from 100 nM to 2 μM at low and high dose tamoxifen respectively. Importantly, within each group of patients treated with the same dose of the drug, there was a high (> 10-fold) individual variability of registered tamoxifen levels both in plasma and in breast tissue (e.g. at 20 mg daily,

the drug concentration varied from 1 to 4 μM in normal breast tissue, and from 0.56 to 6.7 μM in cancerous samples taken from different patients).

In our study the majority of dose-response curves were generated for the range of tamoxifen concentrations from 0.001 to 3 μM.

DNA ERE (D_t)

Identification of the number of direct genomic targets of ER is a difficult task. Moreover this number is likely to be cell type specific and may depend on the cellular microenvironment. Quantifying genome-wide expression levels after estradiol stimulation helps to reveal which genes are regulated by estradiol, however, without discriminating between direct and indirect targets. In this context Chromatin immunoprecipitation (ChIP) is more promising technique. (Welboren *et al*, 2007) provide a review of recent studies focused on identifying ER binding target genes, with the use of ChIP approaches. Existing genome-wide estimates for MCF-7 cells range from 153 promoters bound by ER in the presence of estradiol (Laganiere et al., 2005b) to 578 high-confidence promoter ER targets, of which only 54 were highly responsive to estradiol (Kwon *et al*, 2007). Caroll et al (Carroll *et al*, 2006) report even higher numbers - 3665 unique ER binding sites. Importantly, most existing studies are restricted to MCF-7 cell line. Ross-Innes et al (Ross-Innes *et al*, 2012) were the first to interrogate ER binding events in primary breast cancer samples. With the use of ChiP-seq technology they found a core set of 484 ER binding events that were identified in at least 75% of all the ER-positive tumours.

Thus the majority of existing estimates fall into the range from 100 to 600 promoters per cell, which are directly regulated by ER. Basing on this, and assuming that average volume of MCF-7 cell is 3pL, the concentration of ER-binding promoters is likely to vary from 0.05 nM (100 promoters per cell) to 0.33 nM (600 ER-binding promotors). If to use the highest estimate of 3665 ER binding sites (Carroll *et al*, 2006) the upper constraint can be substantially higher (up to 2 nM).

Therefore in our study we explored our model behavior in the range of Dt concentrations 0.01-1 nM.

Estimation of the rate constants for protein and mRNA synthesis and degradation

k_{dr} was estimated basing on the assumption of half life of mRNA degradation of 7 min. Similar estimates of 4-11 min for mRNA half-life were obtained in (Iyer and Struhl, 1996) for yeast. Genome-wide analysis of RNA transcription and degradation rates in mammalian cells (Rabani *et al*, 2011) also revealed that a median half life of RNA was approx 30 min, with many genes having half-lives shorter than 10 min. k_{sp} =0.001 was based on the estimate of approx. 7 proteins per mRNA per hour, which is in agreement with the data reported in (Schwanhausser *et al*, 2011), where a median translation rate constant was estimated as about 40 proteins per mRNA per hour, and less than 10 proteins per mRNA per hour for less abundant proteins. Finally, k_{dp} $=2*10^{-5}$ was estimated based on the assumption of average protein half-life about 10-20 h for unstable proteins (Schwanhausser *et al*, 2011)

References

Carroll JS, Meyer CA, Song J, Li W, Geistlinger TR, Eeckhoute J, Brodsky AS, Keeton EK, Fertuck KC, Hall GF, Wang Q, Bekiranov S, Sementchenko V, Fox EA, Silver PA, Gingeras TR, Liu XS, Brown M (2006) Genome-wide analysis of estrogen receptor binding sites. *Nat Genet* **38:** 1289-1297.

Iyer V, Struhl K (1996) Absolute mRNA levels and transcriptional initiation rates in Saccharomyces cerevisiae. *Proc Natl Acad Sci U S A* **93:** 5208-5212.

Kisanga ER, Gjerde J, Guerrieri-Gonzaga A, Pigatto F, Pesci-Feltri A, Robertson C, Serrano D, Pelosi G, Decensi A, Lien EA (2004) Tamoxifen and metabolite concentrations in serum and breast cancer tissue during three dose regimens in a randomized preoperative trial. *Clin Cancer Res* **10:** 2336-2343.

Kwon YS, Garcia-Bassets I, Hutt KR, Cheng CS, Jin M, Liu D, Benner C, Wang D, Ye Z, Bibikova M, Fan JB, Duan L, Glass CK, Rosenfeld MG, Fu XD (2007) Sensitive ChIP-DSL technology reveals an extensive estrogen receptor alpha-binding program on human gene promoters. *Proc Natl Acad Sci U S A* **104:** 4852-4857.

Lonning PE, Haynes BP, Straume AH, Dunbier A, Helle H, Knappskog S, Dowsett M (2011) Recent data on intratumor estrogens in breast cancer. *Steroids* **76:** 786-791.

Olea-Serrano N, Devleeschouwer N, Leclercq G, Heuson JC (1985) Assay for estrogen and progesterone receptors of breast cancer cell lines in monolayer culture. *Eur J Cancer Clin Oncol* **21:** 965-973.

Punnonen R, Lukola A, Kudo R (1984) Cytoplasmic estrogen receptor concentrations in the endometrium of Finnish and Japanese women. *Eur J Obstet Gynecol Reprod Biol* **17:** 321-325.

Rabani M, Levin JZ, Fan L, Adiconis X, Raychowdhury R, Garber M, Gnirke A, Nusbaum C, Hacohen N, Friedman N, Amit I, Regev A (2011) Metabolic labeling of RNA uncovers principles of RNA production and degradation dynamics in mammalian cells. *Nat Biotechnol* **29:** 436-442.

Reddel RR, Murphy LC, Hall RE, Sutherland RL (1985) Differential sensitivity of human breast cancer cell lines to the growth-inhibitory effects of tamoxifen. *Cancer Res* **45:** 1525-1531.

Ross-Innes CS, Stark R, Teschendorff AE, Holmes KA, Ali HR, Dunning MJ, Brown GD, Gojis O, Ellis IO, Green AR, Ali S, Chin SF, Palmieri C, Caldas C, Carroll JS (2012) Differential oestrogen receptor binding is associated with clinical outcome in breast cancer. *Nature*.

Schwanhausser B, Busse D, Li N, Dittmar G, Schuchhardt J, Wolf J, Chen W, Selbach M (2011) Global quantification of mammalian gene expression control. *Nature* **473:** 337-342.

Vona G, Sabile A, Louha M, Sitruk V, Romana S, Schutze K, Capron F, Franco D, Pazzagli M, Vekemans M, Lacour B, Brechot C, Paterlini-Brechot P (2000) Isolation by size of epithelial tumor cells : a new method for the immunomorphological and molecular characterization of circulatingtumor cells. *Am J Pathol* **156:** 57-63.

Welboren WJ, Stunnenberg HG, Sweep FC, Span PN (2007) Identifying estrogen receptor target genes. *Mol Oncol* **1:** 138-143.
