# Supplementary figures and images for: A model of estrogen-related gene expression reveals non-linear effects in transcriptional response to tamoxifen
Source: BMC Syst Biol. 2012 Nov 8;6:138. doi: 10.1186/1752-0509-6-138 (PMC3573949; doi:10.1186/1752-0509-6-138)

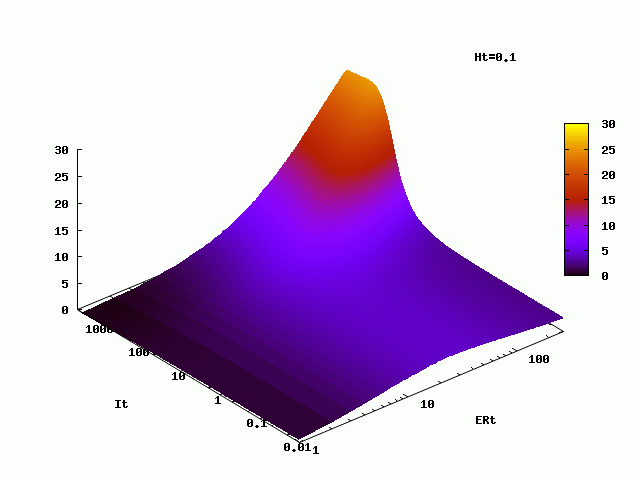

Supplement: Additional file 5 — An animation, demonstrating how the 3D graph shown in Figure 6C evolves with gradual increase in background estradiol concentration. [file 1752-0509-6-138-S5.gif]
