# Supplementary material for: A model of estrogen-related gene expression reveals non-linear effects in transcriptional response to tamoxifen
Source: BMC Syst Biol. 2012 Nov 8;6:138. doi: 10.1186/1752-0509-6-138 (PMC3573949; doi:10.1186/1752-0509-6-138)
Supplement: Additional file 6 — The list of parameters and parameter constraints for GSA [file 1752-0509-6-138-S6.pdf]

Additional File 6

The list of parameters and parameter constraints for GSA

The parameter constraints for GSA were chosen based on biologically plausible assumptions about natural variability of each of the parameters and on available estimates from experimental studies (see Additional File 4).

Parameter	Distribution	Central value	σ
K_1	Gaussian	25.0	1.0
K_2	Gaussian	0.2	0.04
K_3	Gaussian	0.1	0.02
K_4	Gaussian	0.25	0.02

Parameter	Distribution	Lower limit	Upper limit
K_7	Uniform	1.0	50
K_8	Uniform	50	300
K_9	Uniform	26	100
K_{13}	Uniform	0.1	10
K_{15}	Uniform	2.0	17
K_{16}	Uniform	1.0	3.0
K_{17}	Uniform	1.0	3.0
K_{18}	Uniform	10	100
K_{19}	Uniform	20	120
K_{20}	Uniform	10	100
$k_{sr,h}$	Uniform	0.05	0.5
$k_{sr,hi}$	Uniform	0.01	0.1
$k_{sr,i}$	Uniform	0.01	0.1
$k_{sr,b}$	Uniform	0.0005	0.05
H_t	Uniform	0.1	1.0
D_t	Uniform	0.1	1.0
ER_t	Uniform	1.0	200.0

For the majority of the model parameters we sampled the values from the uniform distribution between lower and upper limits, with the exception of K_1, K_2, K_3, and K_4 , whose values were sampled from the Gaussian distribution. This is due to the fact that dissociation constants K_1-K_4 correspond to the binding of natural hormone 17β-estradiol with ER and supposedly their values are subject to less biological variability than the rest of model parameters. In addition to that, the available data set (see Fig.2 in the main text) allowed more reliable identification of these values, as compared to the rest of model parameters.
